# Patient Safety Incidents Involving Sick Children in Primary Care in England and Wales: A Mixed Methods Analysis

**DOI:** 10.1371/journal.pmed.1002217

**Published:** 2017-01-17

**Authors:** Philippa Rees, Adrian Edwards, Colin Powell, Peter Hibbert, Huw Williams, Meredith Makeham, Ben Carter, Donna Luff, Gareth Parry, Anthony Avery, Aziz Sheikh, Liam Donaldson, Andrew Carson-Stevens

**Affiliations:** 1 Division of Population Medicine, Cardiff University, Cardiff, United Kingdom; 2 Institute of Child Health, University College London, London, United Kingdom; 3 Australian Institute for Healthcare Innovation, Macquarie University, Macquarie, Australia; 4 Department of Biostatistics and Health Informatics, Institute of Psychiatry, Psychology and Neuroscience, King’s College London, London, United Kingdom; 5 Institute for Professionalism and Ethical Practice, Boston Children’s Hospital, Boston, Massachusetts, United States of America; 6 Department of Anesthesia, Boston Children’s Hospital, Boston, Massachusetts, United States of America; 7 Harvard Medical School, Harvard University, Boston, Massachusetts, United States of America; 8 Institute for Healthcare Improvement, Cambridge, Massachusetts, United States of America; 9 Division of General Practice, University of Nottingham, Nottingham, United Kingdom; 10 Usher Institute of Population Health Sciences and Informatics, University of Edinburgh, Edinburgh, United Kingdom; 11 Department of Non-communicable Disease Epidemiology, London School of Hygiene and Tropical Medicine, London, United Kingdom; 12 Department of Family Practice, University of British Columbia, Vancouver, British Columbia, Canada; RAND Corporation, UNITED STATES

## Abstract

**Background:**

The UK performs poorly relative to other economically developed countries on numerous indicators of care quality for children. The contribution of iatrogenic harm to these outcomes is unclear. As primary care is the first point of healthcare contact for most children, we sought to investigate the safety of care provided to children in this setting.

**Methods and Findings:**

We undertook a mixed methods investigation of reports of primary care patient safety incidents involving sick children from England and Wales’ National Reporting and Learning System between 1 January 2005 and 1 December 2013. Two reviewers independently selected relevant incident reports meeting prespecified criteria, and then descriptively analyzed these reports to identify the most frequent and harmful incident types. This was followed by an in-depth thematic analysis of a purposive sample of reports to understand the reasons underpinning incidents. Key candidate areas for strengthening primary care provision and reducing the risks of systems failures were then identified through multidisciplinary discussions.

Of 2,191 safety incidents identified from 2,178 reports, 30% (*n* = 658) were harmful, including 12 deaths and 41 cases of severe harm. The children involved in these incidents had respiratory conditions (*n* = 387; 18%), injuries (*n* = 289; 13%), nonspecific signs and symptoms, e.g., fever (*n* = 281; 13%), and gastrointestinal or genitourinary conditions (*n* = 268; 12%), among others. Priority areas for improvement included safer systems for medication provision in community pharmacies; triage processes to enable effective and timely assessment, diagnosis, and referral of acutely sick children attending out-of-hours services; and enhanced communication for robust safety netting between professionals and parents. The main limitations of this study result from underreporting of safety incidents and variable data quality. Our findings therefore require further exploration in longitudinal studies utilizing case review methods.

**Conclusions:**

This study highlights opportunities to reduce iatrogenic harm and avoidable child deaths. Globally, healthcare systems with primary-care-led models of delivery must now examine their existing practices to determine the prevalence and burden of these priority safety issues, and utilize improvement methods to achieve sustainable improvements in care quality.

## Abstract 2

Using a mixed methods approach, Philippa Rees and colleagues describe patient safety incidents involving sick children in primary care in England & Wales.

## Author Summary

**Why Was This Study Done?:**

- Children receive most of their healthcare in the community setting rather than the hospital setting, but very little is known about the safety of this care.
- There are signs from previous research that the UK is providing poorer quality pediatric care than its similarly economically developed counterparts.
- The purpose of this study was to identify what safety concerns there are involving children in primary care, in order to accelerate and inform improvement efforts.

**What Did the Researchers Do and Find?:**

- We analyzed 2,191 reports from a national collection of patient safety incidents that involved sick children in primary care in England and Wales.
- Of the incidents included in this study, 30% were reported as harmful.
- Medication errors, particularly in the community pharmacy setting, were commonly reported.
- Incidents that involved diagnosis, assessment, or referral of sick children were the most harmful of those reported: there were ten deaths, 15 reports of severe harm, and 69 reports of moderate harm.
- Poor communication underpinned many of the safety incidents reported as harming children.

**What Do These Findings Mean?:**

- It is important to note that our findings are limited by the biased nature of incident report data (not all incidents get reported) and require further studies to confirm them.
- However, the frequency with which certain incidents are reported clearly points to areas of care requiring improvement.
- Safer and more reliable medication dispensing systems are needed.
- Out-of-hours telephone triage systems are not fit for pediatric purpose and require improvement.
- Mandatory pediatric training for all general practice trainees is essential.
- We hope that this study acts as an impetus for long-overdue widespread improvement efforts in this area.

## Introduction

The United Kingdom (UK) has one of the highest child mortality rates in Western Europe: the 2,000 excess child deaths that occur annually compare unfavorably with Sweden, which is the best performing country in this region [1–3]. Intercountry variability in rates of child mortality is a well-described global problem. Despite this, there has been a dearth of research on the contribution of unsafe care to these potentially preventable child deaths [4,5].

Primary care is responsible for the majority of healthcare encounters in high-income countries. The safety of care provided to children in this setting is not well understood [6]. For example, in the UK, deaths from meningitis and pneumococcal infection—conditions whose outcomes rely heavily on “first access” services—are considerably higher than in other European countries [3,7,8]. Yet, the avoidable causative factors have not been identified with sufficient clarity for planning action that will prevent the delivery of unsafe care. Furthermore, increasing rates of inappropriate hospital admissions and avoidable referrals to hospital pediatric services indicate that primary care is struggling to meet the demands and changing needs of the pediatric population [7,9–12].

To our knowledge, no systematic approach has been taken to studying the burden of iatrogenic harm in children [4,5,13,14]. Methods that have been used include analysis of vital statistics and case note reviews (some guided by trigger tools) [13–17]. These methods are seldom able to explain why incidents occurred, an essential prerequisite to designing interventions to mitigate future unsafe practice [4]. On the other hand, incident reporting systems can provide detailed descriptions of safety incidents and their underlying contributory factors. Analyses of national repositories of patient safety incident reports have enabled detection and mitigation of rare and serious healthcare safety risks [18–24]. These analyses, in turn, can inform recommendations for clinical process redesign [18–22,25].

This study aimed to characterize the nature and severity of patient safety incidents involving sick children in primary care, to identify potential priority areas requiring action, and to make recommendations for improvement.

## Methods

### Ethical Approval

The Aneurin Bevan University Health Board research risk review committee waived the need for ethics review given the anonymized nature of the data (ABHB R and D reference number SA/410/13), and we therefore did not require informed consent.

### National Reporting and Learning System

The National Reporting and Learning System (NRLS) is a national repository of voluntarily submitted patient safety incident reports from healthcare organizations in England and Wales. Patient safety incidents are defined as “any unintended or unexpected incidents that could have, or did, lead to harm for one or more patients receiving NHS care” [26]. The NRLS was established in 2003 and is the largest repository of its kind, receiving approximately 65,000 reports of patient safety incidents involving children each year [4].

Healthcare professionals submit reports to their local healthcare organizations, where the reports are first analyzed and anonymized, and then submitted in batches to the NRLS. Reports can also be submitted directly to the NRLS online [26–28]. Each report captures structured categorical information such as patient age, incident location, incident date, and severity of harm outcome (no harm, low harm, moderate harm, severe harm, or death) [26–28]. In addition, each report contains three unstructured free-text fields where reporters can describe what happened, why they think it happened, and how they think it could have been prevented [26–28].

### Sample Selection

**Fig 1:**
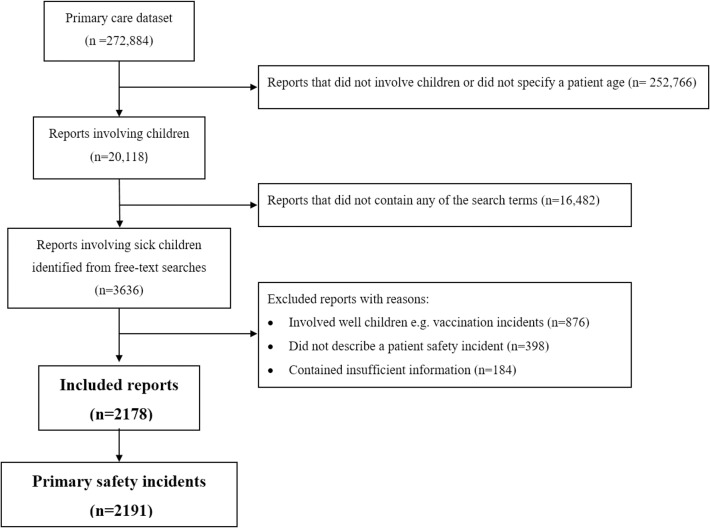
A flow diagram illustrating how reports were selected, included, and excluded.

All incident reports submitted to the NRLS between 1 January 2005 and 1 December 2013 from primary care and involving sick children less than 18 y old were included. Primary care refers to generalist care in the community including, but not limited to, care provided by general practitioners (GPs) (or family physicians), community nurses, and community pharmacists. Reports involving sick children were broadly defined as any reports with descriptions of diagnoses, signs, symptoms, or prescribed medications implying acute or chronic illness in a child. Reports involving children were identified through applying an age filter, and reports involving sick children were identified through free-text searches using key terms and related permutations (Fig 1; S1 Text).

### Methodology

A retrospective cross-sectional mixed methods study was conducted. This involved systematically coding data using multiple coding frameworks to describe the incident, quantitatively exploring coded data to identify important patterns, and thematically analyzing a purposive sample of reports containing new theoretical insights. This methodology has been accepted by the international literature [23,25,28].

### Data Coding

Each incident report underwent data coding using multi-axial frameworks to describe incident types (primary and contributory), potential contributory factors, incident outcomes, and harm severity (S2–S4 Texts) [23,25,28]. Primary incidents included those proximal (chronologically) to the patient outcome, whereas contributory incidents included those that contributed to the occurrence of another incident. Multiple codes for incident type, contributory factor, and incident outcome were applied to each report where necessary. The codes were applied systematically and chronologically according to nine recursive incident analysis rules developed by the Australian Patient Safety Foundation (S1 Table) [29]. This permitted modeling of the steps preceding and leading to primary incidents, e.g., contributory incidents and factors, which, in turn, resulted in patient outcomes (S1 Fig). The incident type, contributory factor, and incident outcome frameworks were developed in house [28]. Each incident report in the NRLS comes with a reporter-allocated harm severity; however, where the free-text descriptions conflicted with the reporter-allocated harm severity, harm severity was reclassified using WHO International Classification for Patient Safety definitions (see Table 1 for WHO definitions of harm severity) [3,23,25,30]. The medications involved in medication incidents were recorded and classified using the British National Formulary for Children, and the types of conditions affecting these children were recorded and classified using the International Classification of Diseases (ICD-10) (S2 Table) [31,32]. A random 20% sample of reports was independently double-coded by P. R. and H. W.

**Table 1:** Primary incident types described within included incident reports and their associated severity of harm.

Primary Incident Type	Severity of Harm					*N* (Percent) Harmful Incidents	*N* Primary Incidents
	No Harm	Low Harm	Moderate Harm	Severe Harm	Death		
**Medication**	459	143	64	6	2	215 (32%)	674
Dispensing	299	69	17	1	—	87 (23%)	386
Administering	75	29	18	1	—	48 (39%)	123
Prescribing	51	12	4	1	—	17 (25%)	68
Clinical treatment decision	26	22	14	2	2	40 (61%)	66
Other	8	11	11	1	—	23 (74%)	31
**Diagnosis and assessment**	344	50	37	9	9	105 (23%)	449
Inadequate triaging	216	13	1	—	2	16 (7%)	232
Delayed assessment	65	13	9	1		23 (26%)	88
Diagnosis	9	14	14	6	2	36 (80%)	45
Insufficient assessment (nonspecific)*	16	5	3	—	1	9 (36%)	25
Inadequate discharge planning	10	3	5	1	1	10 (50%)	20
Inadequate history taking	18	1	1	—	—	2 (10%)	20
Failure to identify high-risk children	4	—	1	—	2	3 (43%)	7
Inadequate examination	3	1	2			3 (50%)	6
Other	3	—	1	1	1	3 (50%)	6
**Administrative**	179	27	13	3	0	43 (19%)	222
Transfer of patient information	105	16	7	—	—	23 (18%)	128
Access to care	56	7	5	3	—	15 (21%)	71
Appointment management	13	2	1	—	—	3 (19%)	16
Other	5	2	—	—	—	2 (29%)	7
**Referral**	135	36	32	6	1	75 (36%)	210
Delayed referral	79	17	15	4	—	36 (31%)	115
Failure to refer when appropriate	22	6	11	2	1	20 (48%)	42
Inappropriate/incomplete referral	24	8	5	—	—	13 (35%)	37
Referral administrative issue	9	5	1	—	—	6 (40%)	15
Failure to arrange follow-up	1					0 (0%)	1
**Communication**	144	20	11	2	0	33 (19%)	177
Communication with patients/caregivers	127	17	10	2	—	29 (19%)	156
Communication between professionals	17	3	1	—	—	4 (19%)	21
**Treatment and procedures**	53	60	26	7	—	93 (64%)	146
**Equipment**	71	13	5	—	—	18 (20%)	89
**Documentation**	70	2	—	—	—	2 (3%)	72
**Other**	21	19	18	—	—	37 (64%)	58
**Investigations**	30	11	3	—	—	14 (32%)	44
**Transport/transfer of patients**	27	6	9	1	—	16 (37%)	43
**Total**	1,533	387	218	41	12	658 (30%)	2,191

Definitions of harm (from the WHO International Classification for Patient Safety [30]): *no harm*—patient outcome is not symptomatic, and no treatment is required; *low harm*—patient outcome is symptomatic, symptoms are mild, loss of function or harm is minimal or intermediate but short term, and no or minimal intervention is required; *moderate harm—*patient outcome is symptomatic, requiring intervention or an increased length of stay or causing permanent or long-term harm or loss of function; *severe harm—*patient outcome is symptomatic, requiring life-saving intervention or major surgical/medical intervention, shortening life expectancy, or causing major permanent or long-term harm or loss of function; *death***—**on balance of probabilities, death was caused or brought forward in the short term by the incident.

*Described with insufficient detail for further classification.

### Data Analysis

We undertook exploratory descriptive analysis of coded data [33]. The relationships between codes were explored using frequency distributions and cross-tabulations, to identify prevalent patterns in associated incidents and contributory factors (S3 and S4 Tables) [34]. Priority areas were identified based on the frequency and associated severity of harm. Recommendations for addressing these priority areas were informed by the factors reported as contributing to incidents, by focused searches of the literature, and by consultation with subject matter experts [23,25,28].

### Thematic Analysis

A purposive sample of reports that corroborated or contradicted emerging theories or contained “new” insights was identified during data coding [35–37]. These reports were exported for qualitative data analysis (NVivo 9, QSR International), and reread for familiarization. New codes were created to capture additional semantic (descriptive and superficial) insights and latent (underlying or inferred) insights present in reports and the contexts in which incidents occurred [25,35,36]. These codes were grouped into themes and sub-themes (by P. R. and A. C-S.) to support our understanding of the data and the underlying reasons for certain incidents [25,35,36].

## Results

### Overview

Of the 3,636 incident reports potentially involving sick children identified through free-text searches, 2,178 were included; excluded reports involved well children (*n* = 876), did not describe a patient safety incident (*n* = 398), or contained insufficient information for coding (*n* = 184) (Fig 1). Cohen’s kappa (*k*) statistic of inter-rater (coding) reliability for primary incidents was high, *k* = 0.72 (95% CI 0.68–0.75; *p* < 0.01).

**Fig 2:**
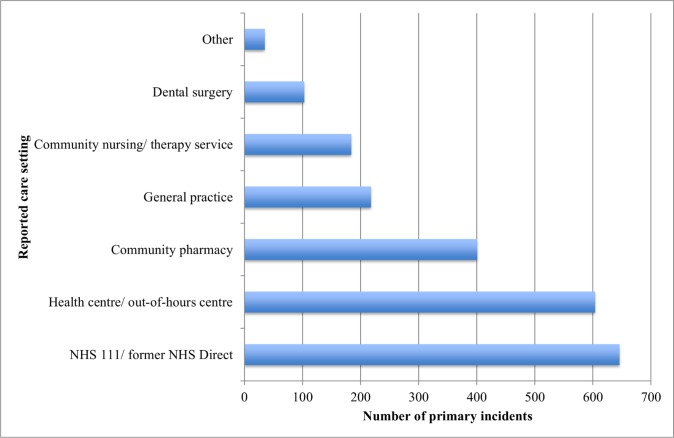
Settings where reported primary-care-related incidents involving sick children occurred. NHS 111 is the UK national telephone triage service.

The incident reports involved care from the UK national telephone triage service, NHS 111 (formerly NHS Direct) (*n* = 646; 30%), out-of-hours health centers (*n* = 604; 28%), community pharmacies (*n* = 401; 18%), and general practices (*n* = 218; 10%) (Fig 2). The 2,178 reports described 2,191 primary incidents (hence 2,191 incidents referred to henceforth), largely involving infants between 28 d and 1 y old (*n* = 491; 22%) and preschool children less than 5 y old (*n* = 542; 25%). The most frequently described conditions included respiratory conditions (*n* = 387; 18%), injuries (*n* = 289; 13%), nonspecific signs and symptoms such as fever (*n* = 281; 13%), and gastrointestinal or genitourinary conditions (*n* = 268; 12%) (Table 2). Included reports described harm to 30% (*n* = 658) of children, including 12 deaths, 41 reports of severe harm, 218 reports of moderate harm, and 387 reports of low harm (Table 1).

**Table 2:** Conditions described in children experiencing safety incidents.

Type of Condition	*N* Primary Incidents
**Respiratory conditions**	387*
Cough, dyspnea, tachypnea, wheezing	127
Asthma	123
Respiratory infection	76
Other	69
**Injuries**	289*
Head injury	123
Poisoning/overdose—accidental or of undetermined intent	42
Limb injury	38
Burn or corrosion	28
Other	60
**Nonspecific signs and symptoms**	281*
Fever	133
Altered consciousness, behavior, or emotions	77
Reduced food and fluid intake/weight loss/failure to thrive	44
**Gastrointestinal or genitourinary conditions**	268*
Disorder of the oral cavity, salivary gland, or jaw	74
Vomiting	69
Abdominal pain	32
Disorder of the stomach, esophagus, or duodenum	22
Genitourinary disorder	21
Other	69
**Skin or musculoskeletal conditions**	245*
Rash	79
Altered skin color	76
Other	91
**Neurological or sensory conditions**	231*
Epilepsy	126
Ear or eye disorder	61
Cerebral palsy or paralytic syndrome	18
Other	34
**Mental or behavior disorders**	221*
Nonspecific mental health issue	65
Intentional self-harm	59
Behavior or emotional disorder with onset in childhood and adolescence	34
Disorder of psychological development	29
Mood disorder	21
Other	20
**Infections**	201
Nonspecific infection	116
Intestinal infectious disease	49
Viral infection characterized by skin and mucous membrane lesions	12
Other	24
**Endocrine, metabolic, or nutrition disorders**	116*
Diabetes mellitus	72
Metabolic disorder	24
Other	21
**Pregnancy/chromosomal or other congenital conditions**	67*
**Cancer and blood conditions**	52*
**Other conditions**	51
**Circulatory system conditions**	50*
**Total**	2,459

*Some children had multiple similar conditions, signs, or symptoms.

Eleven categories of incident types (see Table 1) were evident from included reports. We present a summary of findings related to the priority areas requiring improvement; these include incident types with the highest burden of reported harm in terms of frequency, clinical harm outcomes, and level of harm severity. These priority areas, in descending order of frequency include the unsafe provision of medication, inadequate diagnosis and assessment, and failure of communication with and about the patient (Table 1). Contributory factors for all incidents are summarized in Table 3.

**Table 3:** The contributory factors underpinning reported incidents.

Contributory Factor—*Definition*	*N* Reports
**Staff factors**	722*
Failure to follow protocol—*not adhering to organizational guidelines*	356*
Mistakes—*unintentional cognitive lapses*	272*
Critical thinking—*perception*, *learning*, *memory*, *concept formation*, *problem solving*, *and thinking*	96
Knowledge—*insufficient knowledge or inadequate application of knowledge*	94
Other	11
**Organizational factors**	463*
Continuity of care—*issues with the coordination of services*	149*
Working conditions—*factors relating to the work environment*	148*
Inadequate protocol/guidelines/care plan—*existing guidelines not fit for purpose*	98
Education and training—*insufficient education and training of staff*	74
Service availability—*service inaccessible to patients in a timely manner*	47
Nonspecific	2
**Patient factors**	298*
Age—*age-specific factors*, *e*.*g*., *weight-based dosing*	116
Behavior—*the way in which patients or caregivers act or conduct themselves*	58
Health—*factors relating to the patient’s physical and mental well-being*	55
Geography—*the area where patients live*	38
Knowledge—*insufficient knowledge or inadequate application of knowledge*	30
Language—*patient or caregiver unable to communicate in English*	14
Looked-after—*children not in the care of their parents*, *e*.*g*., *in foster care*	8
Ethnicity—*the patient belongs to a certain social group*	1
**Equipment/medication factors—*the equipment or medication is impractical*, *inadequate*, *or faulty***	78
**Environmental factors—*the physical environment is detrimental to healthcare***	4
**Total**	1,785

*Some reports described multiple contributory factors, e.g., more than one type of mistake.

### Treatment of Sick Children with Medication

The 674 medication-related incidents (primary and contributory; harmful and nonharmful) were described in the home (e.g., from NHS 111 service calls), general practice, and community pharmacy settings. Most incidents (*n* = 386; 57%) were related to dispensing errors in community pharmacies; other medication incidents were administration errors (*n* = 123; 18%) typically in the home setting, prescribing errors (*n* = 68; 10%) in the general practice setting, and clinical treatment decision-making incidents (*n* = 66; 10%) in the general practice or out-of-hours setting (Table 1).

Children less than 1 y old were most frequently (*n* = 131; 19%) involved in reported medication-related incidents, and these children were largely being treated for epilepsy, asthma, and infections (Table 4). As highlighted in Table 4, inhalers for asthma treatment were frequently involved in medication-related incidents: for example, children were dispensed the wrong dose inhaler (*n* = 27), the wrong brand (*n* = 18), or the wrong inhaler medication (*n* = 16). Children with epilepsy were frequently dispensed the wrong dose of anticonvulsant (*n* = 27) or dispensed anticonvulsants with the wrong instruction labels (*n* = 11). Errors involving antimicrobial treatment were related to dispensing the wrong dose (*n* = 13), the wrong medication (*n* = 22), or medications with incorrect labels (*n* = 13).

**Table 4:** Medications involved in medication-related incidents.

Medication Class	Severity of Harm					*N* Primary Incidents (Percent Harmful)
	No Harm	Low Harm	Moderate Harm	Severe Harm	Death	
**Central nervous system**	144*	38	29	3	1	215* (33%)
Antiepileptic	67	13	12	2	—	94
Antipsychotic	21	4	8	1	—	34
Analgesic	21	5	5	—	—	31
Antidepressant	19	8	2	—	—	29*
Other	15	5	2	—	1	23*
Attention deficit hyperactivity disorder medication	12	3	—	—	—	15
**Respiratory system**	125*	20	12*	—	—	157* (20%)
Inhaled corticosteroid	77	10	2	—	—	89
Bronchodilator	28	3	2	—	—	33
Antihistamine, immunotherapy, allergic emergencies	14	5	7	—	—	26
Other	10	2	2	—	—	14
**Infection**	97*	45*	7	1		150* (35%)
Beta-lactam	57	18	—	—	—	75*
Nonspecific antibiotic	10	11	4	—	—	25*
Macrolide	15	7	2	—	—	24
Antiviral	11	5	—	1	—	17
Other	6	8	1	—	—	15
**Endocrine system**	24	6	5	—	—	35 (31%)
**Gastrointestinal system**	13	6	2	—	—	21 (38%)
**Cardiovascular system**	8	2	3	1	—	14 (43%)
**Ear, nose, and oropharynx**	9	4	—	—	—	13 (31%)
**Eye**	8	3	2	—	—	13 (39%)
**Skin**	12	—	1	—	—	13 (8%)
**Musculoskeletal and joint system**	8	2	—	—	—	10 (20%)
**Nutrition and blood**	4	4	—	—	—	8 (50%)
**Anesthesia**	1	2	4	—	—	7 (86%)
**Obstetrics, gynecology, and urinary tract**	3	—	—	—	—	3 (0%)
**Malignant disease and immunosuppression**	—	2	—	—	—	2 (50%)
**Other**	1	—	—	—	1	2 (100%)
**Total**	451*	134*	59*	5*	2	650* (31%)

*Some incidents involved multiple medications, and some did not specify which medications were involved.

Harm resulted from about one-third (*n* = 215; 32%) of medication-related incidents, including two deaths, six reports of severe harm, 64 reports of moderate harm, and 143 reports of low harm (Table 1). Incident outcomes included harm necessitating a hospital visit (*n* = 49), which included admissions to intensive care, e.g., after receiving chlorpromazine rather than chlorphenamine, and deterioration in a child’s condition (*n* = 21), such as increased seizure frequency after dispensing the wrong brand of lamotrigine. In addition, patient inconvenience was a frequently described incident outcome (*n* = 108), such as needing to revisit healthcare professionals (*n* = 52) or experiencing delays in medical management (*n* = 27), e.g., as a result of being dispensed the wrong medication.

Contributory factors were described for most (*n* = 242; 63%) dispensing errors. Staff mistakes were described (*n* = 172), such as confusing medications with similar names or appearances (Examples 1 and 3 in Box 1), e.g., long-acting beta-agonist (LABA) inhalers and LABA/corticosteroid combination inhalers (Example 3). Mistakes occurred in combination with medication factors (*n* = 39), such as different formulations of the same medication having similar packaging, e.g., beclometasone nasal spray and beclometasone inhalers; organizational factors such as busy or distracting work conditions (*n* = 28); or both medication factors and poor working conditions (*n* = 10) (Examples 1, 3, and 4). Other contributing factors included staff failing to follow protocols (*n* = 31), such as preparing two patients’ medications concurrently, and patient age-specific factors (*n* = 23) such as weight-based dose calculation errors (Example 5).

**Box 1. Free-Text Examples of Key Incidents**

These are extracts from the free-text descriptions of incidents provided by the incident reporters. The extracts have been edited by the authors to correct typographical errors and remove indecipherable text.

**Example 1.** Chloramphenicol eye drops 0.5% were prescribed but chloramphenicol ear drops 10% were dispensed from the fridge. This occurred because the medication was dispensed in a hurry and the pharmacist did not spot the error when the second check was made. When the patient used the drops she experienced a prolonged burning sensation and was taken to the hospital when the error was recognised. The different types of chloramphenicol drops had been separated in the past and placed on different shelves due to this error occurring previously. This will now be taken further so that the ear drops are kept in enclosed containers within the fridge and clearly marked on the outside as ear drops. Similar product name. Similar package.

**Example 2.** Dispensing error—prescription for erythromycin 250 mg, dispensed chlorpromazine 50 mg tablets. 16-year-old patient took wrong medicine for 3 days and suffered serious side effects including catatonic seizures. Different brand of chlorpromazine to be kept in pharmacy. Contacted manufacturer to request re-assessment of packaging. Similarity of packaging led to error in tablet selection.

**Example 3.** GP prescribed a 5 year old child chlorphenamine (antihistamine). The pharmacist dispensed chlorpromazine (anti-psychotic) instead of chlorphenamine. Mother did not recognise name so phoned pharmacy to check if it was the same. A member of staff told her that it was the same. Mother gave 8-year-old [sic] 5 ml of 100 mg chlorpromazine. Child became extremely drowsy and was admitted to high dependency unit for observations. Child has since recovered. Pharmacy is reviewing its dispensing procedures and putting these into a written format, i.e., developing standard operating procedures. Poor dispensing procedures and very limited communication between the pharmacist and the patients.

**Example 4.** The prescription read risperidone 1 m/ml dose: 0.25 mg nocte. We supplied the correct product but it was labelled 2.5 ml at night. Although this is a recognised dose for a child of this age it is 10× the prescribed dose. This was a labelling error of unknown cause. The pharmacist did not pick up the labelling error. Additional care needed at time of labelling and checking, especially with children’s prescriptions for unusual medications. Causes: pressure—very busy, interruptions from phone and staff.

**Example 5.** Child of 8 weeks was prescribed ranitidine 75 mg/5 ml. Dose prescribed was 2.5 ml twice a day. Child weighed 3.75 kg. The British National Formulary for Children 2013 indicates that dose should be calculated by weight and from this it was seen that the doctor had prescribed an overdose. The dose should have been 1 mg/kg three times daily. GPs checking the dose in children by weight and weighing the child accurately.

**Example 6.** Baby admitted to Accident and Emergency as sudden unexplained death in infancy aged 2 months having died at home. Baby had been seen by GP on previous evening with temperature of 38 degrees C and possible chest infection, prescribed amoxicillin. NICE [National Institute for Health and Care Excellence] guidance for fever states that fever ≥38 in child less than 3 months is a red flag and a child should be admitted to hospital. Preliminary results from post-mortem suggesting that infection is likely cause of death.

**Example 7.** Patient presented to Accident and Emergency with classical symptoms of new presentation of type 1 diabetes, parents had presented to GP on Friday as concerned he had diabetes—GP recommended further test in 1 week later rather than immediate referral. Parents remained concerned bought blood glucose tester—sugar high. On presentation blood glucose high with 3.3 mmol/l of ketones—blood gas not acidotic. Local & national guidance of immediate referral of all suspected diabetes in children not followed.

**Example 8.** Mum [mother] reporting patient presenting with high temperature, fitting for 2 minutes and drowsiness. Patient has a history of fits. Inappropriate protocol chosen. Should have been assessed under ‘fit’ rather than ‘fever’ as it would have covered all the correct questions and given correct end point.

**Example 9.** Call concerning a baby under 2 months with worsening swelling in umbilical area—baby was crying and had been unwell all day. Nurse advisor used ‘other symptoms’ algorithm instead of unwell baby under 3 month algorithm—she answered 2 questions and then downgraded the call from ‘GP same day’ to ‘GP next working day’. The caller rang back a few hours later and swollen area was worsening, changing colour and baby still crying.

**Example 10.** 4-month-old baby was feverish, had one pupil larger than the other and a hard fontanelle. Call was prioritised as a P2 [the priority allocated to the call after initial triaging]. There was approximately a 20 minute delay before the call was then assessed by a nurse. These symptoms were all potentially very serious so [reporter] called an ambulance without any further assessment. Health advisor used ‘generally unwell’ protocol, and although he asked all the questions he did [not] use any critical thinking when the mother commented that the child was “a little bit more dazed than usual” and “drowsy not with it” and therefore entered the incorrect answer to “are they able to respond normally to you now”. Health advisor commented that he did not know that a hard fontanelle could be dangerous.

**Example 11.** Health advisor answered ‘no’ to a rash that looked like bleeding or bruising when the child did have a mottled purple rash making the call a P3 [the priority allocated to the call after initial triaging]. Health advisor read question addressing ‘does she have a purple discolouration of the skin that looks like bruising or bleeding under the skin’ to which the mother responded ‘no’.

**Example 12.** 10-year-old with injury to arm, swollen and unable to move. Call was placed on queue as P3 [the priority allocated to the call after initial triaging] for three hours. Call back time was given to the caller but no worsening instructions were given. Critical thinking should have been used and clinical advice sought. Health advisor has completed a call reflection and acknowledges she did not give worsening instructions.

**Example 13.** During assessment of call about child with ongoing fever and diarrhea and vomiting, mother informed me that a nurse advisor had given advice yesterday to give ibuprofen and paracetamol at 2 hourly intervals for pain relief. Call listened to. The nurse advisor gave information regarding ibuprofen and paracetamol, but did not say to give them together at 2 hourly intervals. Advice given by the nurse was safe.

Similar contributing factors also underpinned prescribing and administering errors, which often occurred in combination with dispensing errors. For example, most medication administration errors (*n* = 91; 74%) were described as being the result of other incidents, i.e., contributory incidents, typically other medication errors such as dispensing errors (*n* = 41), prescribing errors (*n* = 10), or both (*n* = 7) (see Examples 1 and 3).

### Diagnosis, Assessment, and Referral of Sick Children

The 659 incidents related to diagnosis, assessment, and referral typically occurred in combination and as a result of each other (S3 Table). These incidents occurred via NHS 111 (*n* = 400; 61%), during telephone assessments provided by out-of-hours general practice care (*n* = 158; 24%), or in the general practice setting (*n* = 55; 8%). The children involved were typically young, under 3 y old, and presented acutely with the following: nonspecific signs and symptoms (*n* = 150), particularly fever (*n* = 67) and altered consciousness (*n* = 51); injuries (*n* = 146), particularly head injuries (*n* = 84); and skin or musculoskeletal conditions (*n* = 87), such as rashes (*n* = 34) and skin discoloration (*n* = 33).

Incidents associated with diagnosis, assessment, and referral were the most harmful reported in terms of severity, involving 10 deaths, 15 reports of severe harm, and 69 reports of moderate harm (Table 1). The most frequently described incident outcomes were patient inconvenience (*n* = 179; 27%), particularly as a result of delayed management of conditions (*n* = 157; 24%), and clinical patient harm (*n* = 90; 14%), such as deterioration of a child’s condition (*n* = 43; 7%). Deterioration outcomes also included four cases of potentially fatal diabetic ketoacidosis.

Diagnosis and assessment incidents mostly involved inadequate triaging (*n* = 232; 52%) of acutely unwell children and delayed assessment (*n* = 88; 20%) of these children. Most referral-related incidents (*n* = 154; 73%) involved assessments over the telephone and in the general practice setting, and included delayed referrals (*n* = 115; 55%) and failure to refer a sick child for escalation of care or specialist input when appropriate (*n* = 42; 20%). Incidents contributing to unsafe assessments included the following: inadequate history taking (*n* = 112; 25%); failing to identify high-risk or vulnerable children (*n* = 51; 11%), e.g., those with a history of repeated self-harming; and communication failures, such as inadequate safety netting with parents and caregivers (*n* = 118; 26%). Safety netting is defined within healthcare as providing information (as a safety net) to educate patients, parents, or caregivers and make them aware of when to appropriately seek medical attention in the event of illness, failure to improve, or deterioration medically [38].

Key contributory factors underlying diagnosis, assessment, and referral incidents, particularly those involving inadequate telephone assessments, were related to “protocolized” medicine. Staff failing to follow protocols was frequently described (*n* = 196; 30%), e.g., GPs were described as failing to follow fever and diabetic management guidelines (Examples 6 and 7 in Box 1; Table 3). In the context of telephone assessments, this included non-clinically trained health advisors choosing the wrong protocol, e.g., selecting a “head wound” protocol rather than a “head injury” protocol, or not using the protocol correctly (Examples 8 and 9). Protocols were also described as inadequate (*n* = 35; 5%), e.g., when they led health advisors to underestimate the urgency of the child’s condition. In the context of staff failing to follow protocols, or the protocols failing to adequately assess the urgency of a child’s condition, staff were criticized for not using critical thinking (*n* = 84; 13%; Example 10), despite not having any clinical training.

### Communication Failures with and about the Patient

Of the 177 communication-related incidents reported, 19% (*n* = 33) were harmful, including two reports of severe harm, 11 reports of moderate harm, and 20 reports of low harm (Table 1). Communication failures with patients, parents, and caregivers were described in a range of primary care settings; however, most communication-related incidents occurred either via NHS 111 (*n* = 103; 58%) or in out-of-hours settings (*n* = 39; 22%), and half involved children less than 3 y old (*n* = 90; 51%).

For sick children in primary care, communication failures (*n* = 207) were more commonly reported as contributory rather than as primary incidents. Communication failures frequently underpinned medication incidents, particularly administration errors in the home setting, where parents and caregivers are typically responsible for medication administration, which is influenced by prior communication and instructions from healthcare professionals (Example 3). Communication failures were also frequently implicated in diagnosis and assessment incidents (Example 11), e.g., through inadequate safety netting (Example 12), providing the wrong advice, or not clearly communicating the correct advice (Example 13), particularly with regards to fever management in the context of telephone assessments. The most frequent contributory factor (*n* = 50; 28%) was staff failing to follow protocols, such as those related to safety netting (Table 3).

## Discussion

### Summary

Based on the burden of incidents in terms of their frequency and severity, and the relative contribution of each incident type to subsequent incidents, the primary-care-related priority areas requiring improvement to reduce iatrogenic harm to sick children are the following: medication provision in the community pharmacy setting; telephone assessment and subsequent referral of acutely unwell children; and communication with patients and their caregivers.

### Context of Current Literature

Medication-related safety incidents are widely reported as the most common medical errors, and are thought to be considerably more prevalent in children than in adults [39–42]. Children are more vulnerable to healthcare harm for numerous reasons, such as weight-based dosing; poor availability of certain pediatric formulations, therefore requiring extemporaneous preparation by pharmacists; and dependency on caregivers to advocate for them [5,7,43–45]. Several high-profile reports highlight serious failures in the management of chronic conditions such as asthma and epilepsy in the community setting [2,46–49]. Our study and previous reports highlight that organizational factors (rather than staff knowledge) underpin such failures, suggesting this issue would benefit from quality improvement interventions in healthcare organizations [50–52].

In the UK, children account for 20% of general practice consultations, and 40% of the 500,000 calls received by NHS 111 (formerly NHS Direct) each month [53–56]. Numerous reports in this study criticized telephone assessors for not using critical thinking to challenge inappropriate outcomes reached using clinical decision support (CDS) protocols, arguably due to poor situational awareness. Many have expressed concerns about the safety of telephone assessment of children [53,57–63]. These concerns exist due to the potentially fatal consequences of underestimating the urgency of a child’s condition, the nonspecific nature of many childhood illnesses, the speed with which children deteriorate, and the lack of face-to-face contact, forcing assessors to depend on caregivers to observe the child, interpret those observations, and communicate them effectively [57,59–61,64]. The safety of CDS software used to triage children over the telephone is unclear, particularly its sensitivity to detect signs of serious illness in children [53,60–62,65–68], although its purpose is to minimize risk by standardization and to reduce assessor autonomy—a factor underlying many incidents [61,62].

Despite a study funded by the World Health Organization that echoes our concerns about iatrogenic harm arising from communication failures in primary care, there is a paucity of evaluative studies on this topic, particularly in relation to pediatric telephone assessments [69]. Numerous communication incidents in our study were related to inadequate safety netting during telephone assessment, and this is a well-acknowledged problem in the literature [39,49,70,71]. NHS 111 safety netting protocols have also been described as generic and not child-specific, and there is currently limited evidence to evaluate their role in healthcare-associated harm [38].

### Strengths and Limitations

This is the first national analysis of patient safety incidents focusing on children and young people in the primary care setting, to our knowledge. Exploring problems in primary care as a whole at a national level, and focusing on the combinations of incidents and contributory factors, provides insights into the interaction of factors between various primary care settings that underlie iatrogenic harm and the subsequent trajectory of harm in this heterogeneous setting.

We sought to achieve methodological rigor through independent double-coding of a random 20% sample of reports, weekly meetings to discuss coding, and keeping an audit trail to aid reflexivity [72,73]. Incident report data are limited by underreporting and variable data quality; thus, our findings are not likely to be generalizable. It is not possible to comment on variation in underreporting between incident types or settings, given the unknown true denominator of patient safety incidents in primary care; therefore, we cannot comment on the relative safety of different healthcare settings. However, it is important to note that incident report data provide a considerable body of granular information on incidents and contributory factors perceived to be important by front-line healthcare professionals and staff [41]. In light of this, given the nature of these data, it would be premature to conclude that medication safety is a bigger problem than diagnostic error, or that the GP’s office is a safer care setting than an out-of-hours health center. Longitudinal studies using case note review methods to assess the frequency and burden of unsafe primary care are required to support such claims.

### Recommendations for Improvement

Our recommendations to improve primary care for children are drawn from the literature and were chosen to ensure they specifically target not only the priority areas identified in our study as requiring improvement but also the specific factors described as contributing to incidents in these priority areas. We corroborated our recommendations with subject matter experts.

Community pharmacy dispensing errors could be reduced through electronic transmission of prescriptions from general practice to the dispensing community pharmacy, as this would prevent errors at the prescriber–dispenser interface [74]. We also recommend implementing a bar coding system for all medications (as is often done in hospital pharmacies), to reduce the potential for human error by acting as an additional safety check prior to medication dispensing [75–77]. Education and training of all pharmacy staff in human factors could enable staff to recognize weaknesses in their own practice [78–83]. In addition, building improvement capability among staff could prove an effective and efficient method of improving patient safety [84].

This study supports the UK Royal College of Paediatrics and Child Health’s call for a robust evaluation of the effectiveness of NHS 111 for children and mandatory pediatric training for all general practice trainees [85]. Monitoring the safety of CDS used to triage sick children is a necessity to target improvement efforts to effectively prevent iatrogenic harm to children. Such improvement may include earlier clinician involvement in the assessment of younger children, who are more difficult to triage safely [68,85]. The outcomes of children assessed using CDS should be reviewed, and the CDS software updated and amended to improve its sensitivity and specificity for this population [86–90]. In addition, CDS could be amended to reduce the potential for certain errors, e.g., reminders when triaging head wounds to double-check the absence of a head injury (which would require triaging with a different protocol).

A lack of critical thinking was described as a contributory factor in many telephone triaging incidents. This is a form of poor situational awareness, with situational awareness referring to sensitivity to operations or “knowing what is going on” [91,92]. Examples of how situational awareness could be improved among telephone triaging staff include human factors training, daily safety huddles to provide feedback on positive and negative cases, and encouraging staff to recognize and act when CDS protocols and their outcomes seem inappropriate [86,92–94]. Increasing situational awareness among telephone triaging staff could—in combination with CDS—increase identification of high-risk children and enable mitigation of risks and appropriate escalation of care.

This study’s findings point to a clear need for improved communication with patients, parents, and caregivers in the context of explaining treatment plans, telephone assessments, and providing safety netting via the telephone. Parents and caregivers should receive oral and written information (perhaps via email, text messaging, or smart phone applications, whichever mode they prefer) regarding treatment plans and for safety netting purposes [38]. This approach is currently being rolled out for epilepsy care in the UK in the form of the Epilepsy Passport. In the context of telephone assessments, adherence to safety netting protocols could be improved through the use of mnemonics or checklists [95–98].

### Future Research

In order to expand on our capability to learn from incident report data, higher quality data are needed from healthcare professionals and staff. This will require them to have an understanding of patient safety and human factors, and training to write incident reports [99]. However, to gain a handle on the frequency and burden of unsafe care in children and target improvement efforts, pediatric safety research must mirror the trajectory of ongoing longitudinal studies into the safety of adult care in hospitals and community settings [100,101].

### Conclusion

This study has highlighted opportunities to improve the safety of primary care for children through identifying recurring healthcare failures and commonly reported problems underlying them. Safer, reliable medication dispensing systems, redesigned NHS 111 algorithms that are fit for pediatric purpose, improved situational awareness in triage systems, a deeper understanding of communication failures between parents and primary and secondary care practitioners, and mandatory pediatric training for all general practice trainees are priority areas for redress. Globally, healthcare systems with primary-care-led models of delivery must now examine their existing practices to determine the prevalence and burden of these priority safety issues in care provided to children, in addition to reflecting on our recommendations to address these issues in the context of their own practice.

## Supporting Information

S1 Fig Recursive model of incident analysis. (TIFF)

S1 Table The nine recursive incident analysis rules developed by the Australian Patient Safety Foundation. (DOCX)

S2 Table ICD-10 codes used to classify children’s preexisting and/or presenting conditions. (DOCX)

S3 Table The frequency of combinations of incidents. (DOCX)

S4 Table The frequency of combinations of contributory factors for each primary incident type. (DOCX)

S5 Table STROBE Statement checklist of items that should be included in reports of cross-sectional studies. (DOC)

S6 Table Consolidated Criteria for Reporting Qualitative Research 32-item checklist. (DOCX)

S1 Text Search terms used to retrieve reports involving sick children. (DOCX)

S2 Text Incident types framework. (DOCX)

S3 Text Contributory factors framework. (DOCX)

S4 Text Incident outcomes framework. (DOCX)

S5 Text Research plan. (DOCX)

## Abbreviations

CDS: clinical decision support
GP: general practitioner
NRLS: National Reporting and Learning System
