# Supplementary material for: Patient Safety Incidents Involving Sick Children in Primary Care in England and Wales: A Mixed Methods Analysis
Source: PLoS Med. 2017 Jan 17;14(1):e1002217. doi: 10.1371/journal.pmed.1002217 (PMC5240916; doi:10.1371/journal.pmed.1002217)
Supplement: S1 Table — (DOCX) [file pmed.1002217.s002.docx]

| S1 Table: the nine rules of the Australian Recursive Model of Incident Analysis | |
| --- | --- |
| **Incident Analysis Rules** | **Rule Example** |
| **1. An incident has a set of contributory factors and / or contributory incidents** | Missed diagnosis (incident) because the physician did not adequately examine the patient (contributory incident) and the physician had inadequate knowledge (contributory factor) |
| **2. An incident can contribute to another incident** | Missed diagnosis (contributory incident) resulted in a patient not receiving a timely referral to the hospital (primary incident) |
| **3. Contributory factors cannot be incidents in their own right** | A mistake (contributory factor *not* an incident) resulting in the wrong prescribed medication dose (primary incident) |
| **4. An incident has a set of outcomes** | Wrong prescribed medication dose (primary incident) resulting in a medication overdose and hospital admission (outcomes) |
| **5. An incident can be an outcome of another incident** | Records not up to date (contributory incident) resulting in the wrong prescribed medication (primary incident and outcome) |
| **6. Some outcomes cannot be incidents in their own right** | Admission to hospital (outcome) following the wrong prescribed medication (primary incident) |
| **7. An outcome of an incident could be a contributory incident to another incident** | Communication incident between care providers (contributory incident) resulting in records not being up to date (contributory incident and outcome), resulting in a referral incident (primary incident) |
| **8. An incident can be designated the primary incident type – the incident proximal to the descriptive patient outcome** | Communication incident (incident) leading to inaccurate records (incident), leading to the wrong prescribed medication (primary incident type) |
| **9. The outcome of a primary incident cannot be an incident** | Admission to hospital (outcome) following the wrong prescribed medication dose (primary incident type) |
