# Supplementary material for: Patient Safety Incidents Involving Sick Children in Primary Care in England and Wales: A Mixed Methods Analysis
Source: PLoS Med. 2017 Jan 17;14(1):e1002217. doi: 10.1371/journal.pmed.1002217 (PMC5240916; doi:10.1371/journal.pmed.1002217)
Supplement: S2 Table — (DOCX) [file pmed.1002217.s003.docx]

| **S2 Table:** ICD-10 codes used to classify children’s pre-existing and/ or presenting conditions | |
| --- | --- |
| Type of condition | ICD 10 codes |
| Infections | A00-99; B00-99 |
| Cancer and blood | C00-97; D00-89; R70-79 |
| Skin and musculoskeletal system | L00-99; M00-99; Q65-79; R20-23; R25-29 |
| Neurological and sensory system | G00-99; H00-95; Q00-Q07; Q10-18 |
| Respiratory system | J00-99; Q30-34; R04-6; R09.0-3 |
| Mental and behavior | F00-99; X60-84 |
| Injuries | S00-99; T00-98; V01-X59; X85-Y09; Y10-Y34; Y35-36; Y40-84; Y85-89 |
| Circulatory system | Q20-28; R00-03; R09.8 |
| Digestive and genitourinary system | K00-93; N00-99; Q35-37; Q38-45; Q50-56; Q60-64; R10-19; R30-39; R80-82 |
| Endocrine, metabolic and nutrition | E00-90 |
| Non-specific signs and symptoms | R07; R40-46; R47-49; R50-69; R83-89; R90-94; R95-99 |
| Pregnancy, chromosomal other congenital disorders | O00-99; P00-96; Q80-89; Q90-99 |
