# Supplementary material for: Patient Safety Incidents Involving Sick Children in Primary Care in England and Wales: A Mixed Methods Analysis
Source: PLoS Med. 2017 Jan 17;14(1):e1002217. doi: 10.1371/journal.pmed.1002217 (PMC5240916; doi:10.1371/journal.pmed.1002217)
Supplement: S3 Table — (DOCX) [file pmed.1002217.s004.docx]

| **S3 Table: Frequency of combination of incidents** | | | | | |
| --- | --- | --- | --- | --- | --- |
| **Primary incident** | **Contributory incidents** | | | | **Frequency of combination** |
| Inadequate triaging | Inadequate history |  |  |  | 48 |
| Medication dispensing | Medication prescribing |  |  |  | 44 |
| Medication administration | Medication dispensing |  |  |  | 35 |
| Medication dispensing | Medication dispensing |  |  |  | 22 |
| Inadequate triaging | Communication with parents/ patients |  |  |  | 21 |
| Delayed referral | Inadequate triaging |  |  |  | 19 |
| Treatment and procedures | Equipment |  |  |  | 17 |
| Inadequate triaging | Documentation |  |  |  | 16 |
| Delayed referral | Inadequate triaging | Inadequate history |  |  | 13 |
| Inadequate triaging | Communication between professionals |  |  |  | 13 |
| Inadequate triaging | Inadequate history | Communication with parents/ patients |  |  | 12 |
| Medication administration | Communication with parents/ patients |  |  |  | 12 |
| Delayed assessment | Inadequate triaging |  |  |  | 11 |
| Access to care | Transfer of information |  |  |  | 10 |
| Access to care | Documentation |  |  |  | 10 |
| Delayed assessment | Appointment management |  |  |  | 10 |
| Diagnosis | Investigations |  |  |  | 10 |
| Failure to refer when appropriate | Failure to identify at risk/ vulnerable child |  |  |  | 10 |
| Transfer of information | Documentation |  |  |  | 10 |
| Communication with parents/ patients | Documentation |  |  |  | 8 |
| Delayed assessment | Access to care |  |  |  | 8 |
| Communication with parents/ patients | Inadequate triaging |  |  |  | 7 |
| Delayed referral | Failure to identify at risk/ vulnerable child |  |  |  | 7 |
| Failure to refer when appropriate | Inadequate triaging |  |  |  | 7 |
| Medication administration | Medication prescribing |  |  |  | 7 |
| Other medication | Medication dispensing |  |  |  | 7 |
| Communication with parents/ patients | Inadequate history |  |  |  | 6 |
| Documentation | Inadequate history |  |  |  | 6 |
| Equipment | Equipment |  |  |  | 6 |
| Equipment | Other administrative |  |  |  | 6 |
| Inadequate triaging | Inadequate history | Documentation |  |  | 6 |
| Inadequate triaging | Failure to identify at risk/ vulnerable child |  |  |  | 6 |
| Transfer of information | Transfer of information |  |  |  | 6 |
| Treatment and procedures | Equipment |  |  |  | 6 |
| Access to care | Appointment management |  |  |  | 5 |
| Communication with parents/ patients | Transfer of information |  |  |  | 5 |
| Communication with parents/ patients | Failure to identify at risk/ vulnerable child |  |  |  | 5 |
| Delayed assessment | Transfer of information |  |  |  | 5 |
| Delayed referral | Inadequate triaging | Inadequate history | Communication with parents/ patients |  | 5 |
| Delayed referral | Inadequate triaging | Communication with parents/ patients |  |  | 5 |
| Delayed referral | Delayed assessment |  |  |  | 5 |
| Documentation | Communication with parents/ patients |  |  |  | 5 |
| Inadequate triaging | Documentation | Inadequate history |  |  | 5 |
| Incomplete referral | Inadequate triaging |  |  |  | 5 |
| Medication administration | Medication dispensing | Medication prescribing |  |  | 5 |
| Medication administration | Medication dispensing | Medication dispensing |  |  | 5 |
| Medication administration | Equipment |  |  |  | 5 |
| Medication dispensing | Communication with parents/ patients |  |  |  | 5 |
| Transfer of information | Communication between professionals |  |  |  | 5 |
| Transfer of information | Equipment |  |  |  | 5 |
| Communication with parents/ patients | Investigations |  |  |  | 4 |
| Delayed referral | Failure to identify at risk/ vulnerable child |  |  |  | 4 |
| Inadequate discharge planning | Failure to identify at risk/ vulnerable child |  |  |  | 4 |
| Inadequate history | Communication with parents/ patients |  |  |  | 4 |
| Insufficient assessment (non-specific) | Documentation |  |  |  | 4 |
| Other | Equipment |  |  |  | 4 |
| Other | Treatment and procedures |  |  |  | 4 |
| Transfer of patients | Documentation |  |  |  | 4 |
| Treatment and procedures | Appointment management |  |  |  | 4 |
| Treatment and procedures | Transfer of information |  |  |  | 4 |
| Treatment decision | Other medication |  |  |  | 4 |
| Access to care | Documentation | Communication with parents/ patients |  |  | 3 |
| Access to care | Communication with parents/ patients |  |  |  | 3 |
| Communication with parents/ patients | Treatment decision |  |  |  | 3 |
| Delayed assessment | Incomplete referral |  |  |  | 3 |
| Delayed assessment | Communication with parents/ patients |  |  |  | 3 |
| Delayed referral | Inadequate triaging | Documentation |  |  | 3 |
| Delayed referral | Inadequate triaging |  |  |  | 3 |
| Failure to refer when appropriate | Investigations |  |  |  | 3 |
| Inadequate triaging | Documentation | Inadequate history | Communication with parents/ patients |  | 3 |
| Insufficient assessment (non-specific) | Equipment |  |  |  | 3 |
| Investigations | Communication with parents/ patients |  |  |  | 3 |
| Medication dispensing | Medication dispensing | Medication prescribing |  |  | 3 |
| Transfer of information | Communication between professionals |  |  |  | 3 |
| Transfer of information | Appointment management |  |  |  | 3 |
| Transfer of patients | Equipment |  |  |  | 3 |
| Transfer of patients | Insufficient assessment (non-specific) |  |  |  | 3 |
| Treatment and procedures | Treatment and procedures |  |  |  | 3 |
| Treatment decision | Medication dispensing |  |  |  | 3 |
| Treatment decision | Diagnosis |  |  |  | 3 |
| Access to care | Incomplete referral |  |  |  | 2 |
| Access to care | Other administrative |  |  |  | 2 |
| Access to care | Incomplete referral |  |  |  | 2 |
| Appointment management | Communication between professionals |  |  |  | 2 |
| Communication with parents/ patients | Failure to refer when appropriate |  |  |  | 2 |
| Communication with parents/ patients | Incomplete referral |  |  |  | 2 |
| Communication with parents/ patients | Diagnosis | Insufficient assessment (non-specific |  |  | 2 |
| Communication with parents/ patients | Inadequate triaging | Inadequate history |  |  | 2 |
| Communication with parents/ patients | Inadequate triaging | Communication between professionals |  |  | 2 |
| Delayed assessment | Documentation | Transfer of information |  |  | 2 |
| Delayed assessment | Referral administrative issues |  |  |  | 2 |
| Delayed assessment | Inadequate triaging | Communication with parents/ patients |  |  | 2 |
| Delayed assessment | Inadequate discharge planning | Appointment management | Communication with parents/ patients |  | 2 |
| Delayed referral | Transfer of information |  |  |  | 2 |
| Delayed referral | Inadequate triaging | Failure to identify at risk/ vulnerable child |  |  | 2 |
| Delayed referral | Communication between professionals |  |  |  | 2 |
| Delayed referral | Transfer of patients |  |  |  | 2 |
| Delayed referral | Documentation |  |  |  | 2 |
| Delayed referral | Communication with parents/ patients |  |  |  | 2 |
| Delayed referral | Equipment |  |  |  | 2 |
| Diagnosis | Investigations | Failure to identify at risk/ vulnerable child |  |  | 2 |
| Documentation | Other administrative |  |  |  | 2 |
| Documentation | Investigations |  |  |  | 2 |
| Equipment | Inadequate discharge planning |  |  |  | 2 |
| Failure to refer when appropriate | Inadequate history |  |  |  | 2 |
| Failure to refer when appropriate | Transfer of information |  |  |  | 2 |
| Inadequate history | Failure to identify at risk/ vulnerable child |  |  |  | 2 |
| Inadequate triaging | Communication with parents/ patients |  |  |  | 2 |
| Inadequate triaging | Inadequate history | Documentation | Communication with parents/ patients |  | 2 |
| Inadequate triaging | Failure to identify at risk/ vulnerable child | Communication with parents/ patients |  |  | 2 |
| Incomplete referral | Failure to identify at risk/ vulnerable child |  |  |  | 2 |
| Insufficient assessment (non-specific) | Treatment and procedures |  |  |  | 2 |
| Investigations | Transfer of information | Appointment management |  |  | 2 |
| Medication administration | Transfer of information |  |  |  | 2 |
| Medication dispensing | Communication between professionals |  |  |  | 2 |
| Medication dispensing | Communication between professionals |  |  |  | 2 |
| Medication monitoring | Appointment management |  |  |  | 2 |
| Medication prescribing | Documentation |  |  |  | 2 |
| Other | Appointment management |  |  |  | 2 |
| Other | Other |  |  |  | 2 |
| Other | Transfer of information |  |  |  | 2 |
| Other | Access to care |  |  |  | 2 |
| Other medication | Equipment |  |  |  | 2 |
| Treatment decision | Communication with parents/ patients |  |  |  | 2 |
| Treatment decision | Transfer of information |  |  |  | 2 |
| Treatment decision | Inadequate examination |  |  |  | 2 |
| Treatment decision | Medication prescribing |  |  |  | 2 |
| Access to care | Other administrative | Communication with parents/ patients |  |  | 1 |
| Access to care | Transfer of information | Equipment |  |  | 1 |
| Access to care | Transfer of information | Transfer of information |  |  | 1 |
| Access to care | Transfer of information | Other |  |  | 1 |
| Access to care | Inadequate triaging | Inadequate history |  |  | 1 |
| Access to care | Communication with parents/ patients | Documentation |  |  | 1 |
| Access to care | Appointment management | Inadequate triaging | Failure to identify at risk/ vulnerable child |  | 1 |
| Access to care | Equipment |  |  |  | 1 |
| Access to care | Transfer of information | Transfer of information |  |  | 1 |
| Access to care | Appointment management | Inadequate triaging |  |  | 1 |
| Access to care | Transfer of patients |  |  |  | 1 |
| Access to care | Appointment management | Failure to refer when appropriate |  |  | 1 |
| Access to care | Transfer of patients | Communication between professionals |  |  | 1 |
| Access to care | Referral administrative issues |  |  |  | 1 |
| Access to care | Inadequate triaging |  |  |  | 1 |
| Appointment management | Referral administrative issues |  |  |  | 1 |
| Appointment management | Failure to identify at risk/ vulnerable child | Inadequate triaging |  |  | 1 |
| Communication between professionals | Transfer of information | Inadequate discharge planning |  |  | 1 |
| Communication between professionals | Documentation |  |  |  | 1 |
| Communication between professionals | Inadequate history |  |  |  | 1 |
| Communication with parents/ patients | Treatment decision |  |  |  | 1 |
| Communication with parents/ patients | Medication prescribing |  |  |  | 1 |
| Communication with parents/ patients | Inadequate triaging | Access to care | Appointment management |  | 1 |
| Communication with parents/ patients | Other |  |  |  | 1 |
| Communication with parents/ patients | Other | Documentation |  |  | 1 |
| Communication with parents/ patients | Documentation | Inadequate history |  |  | 1 |
| Communication with parents/ patients | Delayed referral | Inadequate triaging |  |  | 1 |
| Communication with parents/ patients | Failure to refer when appropriate | Failure to identify at risk/ vulnerable child |  |  | 1 |
| Communication with parents/ patients | Incomplete referral | Inadequate triaging |  |  | 1 |
| Communication with parents/ patients | Diagnosis | Failure to identify at risk/ vulnerable child |  |  | 1 |
| Communication with parents/ patients | Inadequate triaging | Inadequate history | Other |  | 1 |
| Communication with parents/ patients | Failure to identify at risk/ vulnerable child | Equipment |  |  | 1 |
| Communication with parents/ patients | Failure to identify at risk/ vulnerable child | Inadequate triaging |  |  | 1 |
| Communication with parents/ patients | Insufficient assessment (non-specific | Access to care |  |  | 1 |
| Communication with parents/ patients | Insufficient assessment (non-specific | Equipment | Equipment |  | 1 |
| Communication with parents/ patients | Delayed assessment | Inadequate history |  |  | 1 |
| Communication with parents/ patients | Treatment and procedures |  |  |  | 1 |
| Communication with parents/ patients | Treatment decision | Inadequate examination |  |  | 1 |
| Communication with parents/ patients | Equipment |  |  |  | 1 |
| Communication with parents/ patients | Appointment management |  |  |  | 1 |
| Communication with parents/ patients | Inadequate history |  |  |  | 1 |
| Delayed assessment | Other administrative |  |  |  | 1 |
| Delayed assessment | Appointment management | Transfer of information |  |  | 1 |
| Delayed assessment | Access to care | Appointment management |  |  | 1 |
| Delayed assessment | Access to care | Referral administrative issues |  |  | 1 |
| Delayed assessment | Access to care | Equipment |  |  | 1 |
| Delayed assessment | Access to care | Inadequate triaging |  |  | 1 |
| Delayed assessment | Other | Appointment management |  |  | 1 |
| Delayed assessment | Documentation |  |  |  | 1 |
| Delayed assessment | Delayed referral |  |  |  | 1 |
| Delayed assessment | Delayed referral | Failure to identify at risk/ vulnerable child |  |  | 1 |
| Delayed assessment | Failure to refer when appropriate |  |  |  | 1 |
| Delayed assessment | Referral administrative issues | Communication between professionals |  |  | 1 |
| Delayed assessment | Inadequate triaging | Documentation | Documentation |  | 1 |
| Delayed assessment | Inadequate triaging | Inadequate history |  |  | 1 |
| Delayed assessment | Inadequate triaging | Inadequate history | Communication with parents/ patients |  | 1 |
| Delayed assessment | Inadequate triaging | Failure to identify at risk/ vulnerable child |  |  | 1 |
| Delayed assessment | Inadequate triaging | Communication between professionals |  |  | 1 |
| Delayed assessment | Inadequate triaging | Equipment |  |  | 1 |
| Delayed assessment | Failure to identify at risk/ vulnerable child |  |  |  | 1 |
| Delayed assessment | Failure to identify at risk/ vulnerable child | Communication between professionals |  |  | 1 |
| Delayed assessment | Investigations | Access to care |  |  | 1 |
| Delayed assessment | Communication with parents/ patients | Inadequate triaging |  |  | 1 |
| Delayed assessment | Communication with parents/ patients | Inadequate triaging | Inadequate triaging |  | 1 |
| Delayed assessment | Communication between professionals | Incomplete referral | Access to care | Appointment management | 1 |
| Delayed assessment | Communication between professionals | Incomplete referral | Failure to identify at risk/ vulnerable child |  | 1 |
| Delayed assessment | Equipment |  |  |  | 1 |
| Delayed referral | Failure to arrange follow up | Transfer of information |  |  | 1 |
| Delayed referral | Incomplete referral | Failure to identify at risk/ vulnerable child |  |  | 1 |
| Delayed referral | Diagnosis | Documentation |  |  | 1 |
| Delayed referral | Diagnosis | Diagnosis |  |  | 1 |
| Delayed referral | Access to care |  |  |  | 1 |
| Delayed referral | Other |  |  |  | 1 |
| Delayed referral | Diagnosis | Incomplete referral |  |  | 1 |
| Delayed referral | Insufficient assessment (non-specific) |  |  |  | 1 |
| Delayed referral | Inadequate triaging | Access to care |  |  | 1 |
| Delayed referral | Inadequate triaging | Documentation | Inadequate history |  | 1 |
| Delayed referral | Inadequate triaging | Documentation | Inadequate history | Communication with parents/ patients | 1 |
| Delayed referral | Inadequate triaging | Medication dispensing |  |  | 1 |
| Delayed referral | Inadequate history |  |  |  | 1 |
| Delayed referral | Inadequate examination | Failure to identify at risk/ vulnerable child | Communication with parents/ patients |  | 1 |
| Delayed referral | Delayed assessment | Access to care |  |  | 1 |
| Delayed referral | Delayed assessment | Inadequate triaging |  |  | 1 |
| Delayed referral | Delayed assessment | Inadequate triaging | Documentation |  | 1 |
| Delayed referral | Delayed assessment | Inadequate triaging | Communication between professionals |  | 1 |
| Delayed referral | Treatment decision | Communication with parents/ patients | Failure to identify at risk/ vulnerable child | Inadequate examination | 1 |
| Delayed referral | Investigations | Failure to identify at risk/ vulnerable child |  |  | 1 |
| Delayed referral | Communication with parents/ patients | Inadequate triaging |  |  | 1 |
| Delayed referral | Communication with parents/ patients | Inadequate triaging | Failure to identify at risk/ vulnerable child |  | 1 |
| Delayed referral | Inadequate triaging |  |  |  | 1 |
| Delayed referral | Communication with parents/ patients |  |  |  | 1 |
| Delayed referral | Diagnosis | Documentation |  |  | 1 |
| Diagnosis | Failure to identify at risk/ vulnerable child |  |  |  | 1 |
| Diagnosis | Investigations | Communication between professionals |  |  | 1 |
| Diagnosis | Transfer of information |  |  |  | 1 |
| Diagnosis | Failure to identify at risk/ vulnerable child | Inadequate history | Transfer of information.5 |  | 1 |
| Diagnosis | Failure to identify at risk/ vulnerable child | Investigations |  |  | 1 |
| Diagnosis | Delayed referral | Documentation |  |  | 1 |
| Diagnosis | Investigations | Diagnosis | Investigations |  | 1 |
| Diagnosis | Access to care |  |  |  | 1 |
| Diagnosis | Delayed referral | Failure to identify at risk/ vulnerable child |  |  | 1 |
| Diagnosis | Diagnosis |  |  |  | 1 |
| Diagnosis | Appointment management | Communication with parents/ patients |  |  | 1 |
| Diagnosis | Transfer of information | Other administrative | Other administrative |  | 1 |
| Diagnosis | Delayed referral |  |  |  | 1 |
| Diagnosis | Inadequate examination |  |  |  | 1 |
| Diagnosis | Failure to identify at risk/ vulnerable child |  |  |  | 1 |
| Diagnosis | Investigations | Inadequate history |  |  | 1 |
| Diagnosis | Investigations | Communication between professionals |  |  | 1 |
| Diagnosis | Investigations | Other administrative |  |  | 1 |
| Diagnosis | Communication with parents/ patients |  |  |  | 1 |
| Documentation | Transfer of information |  |  |  | 1 |
| Documentation | Appointment management |  |  |  | 1 |
| Documentation | Equipment |  |  |  | 1 |
| Documentation | Communication between professionals | Transfer of information |  |  | 1 |
| Documentation | Investigations |  |  |  | 1 |
| Documentation | Other | Other |  |  | 1 |
| Documentation | Insufficient assessment (non-specific) |  |  |  | 1 |
| Documentation | Inadequate history | Communication with parents/ patients |  |  | 1 |
| Documentation | Inadequate history | Communication with parents/ patients | Communication with parents/ patients |  | 1 |
| Documentation | Inadequate examination |  |  |  | 1 |
| Documentation | Insufficient assessment (non-specific) | Appointment management |  |  | 1 |
| Documentation | Communication with parents/ patients | Inadequate history |  |  | 1 |
| Equipment | Medication dispensing |  |  |  | 1 |
| Equipment | Equipment | Referral administrative issues | Incomplete referral |  | 1 |
| Equipment | Access to care | Transfer of information |  |  | 1 |
| Equipment | Failure to refer when appropriate |  |  |  | 1 |
| Equipment | Inadequate discharge planning | Transfer of information |  |  | 1 |
| Equipment | Medication prescribing |  |  |  | 1 |
| Equipment | Other medication |  |  |  | 1 |
| Equipment | Transfer of information |  |  |  | 1 |
| Equipment | Delayed assessment |  |  |  | 1 |
| Failure to arrange follow up | Delayed assessment |  |  |  | 1 |
| Failure to identify at risk/ vulnerable child | Inadequate triaging | Inadequate history |  |  | 1 |
| Failure to refer when appropriate | Investigations | Investigations |  |  | 1 |
| Failure to refer when appropriate | Access to care | Appointment management |  |  | 1 |
| Failure to refer when appropriate | Transfer of information | Communication between professionals |  |  | 1 |
| Failure to refer when appropriate | Inadequate triaging | Inadequate history | Communication with parents/ patients |  | 1 |
| Failure to refer when appropriate | Inadequate triaging | Failure to identify at risk/ vulnerable child |  |  | 1 |
| Failure to refer when appropriate | Communication with parents/ patients |  |  |  | 1 |
| Failure to refer when appropriate | Communication with parents/ patients | Failure to identify at risk/ vulnerable child |  |  | 1 |
| Failure to refer when appropriate | Communication between professionals |  |  |  | 1 |
| Inadequate discharge planning | Transfer of information | Access to care |  |  | 1 |
| Inadequate discharge planning | Documentation |  |  |  | 1 |
| Inadequate discharge planning | Failure to refer when appropriate |  |  |  | 1 |
| Inadequate discharge planning | Incomplete referral |  |  |  | 1 |
| Inadequate discharge planning | Communication between professionals | Transfer of information |  |  | 1 |
| Inadequate discharge planning | Communication between professionals | Transfer of information |  |  | 1 |
| Inadequate discharge planning | Equipment |  |  |  | 1 |
| Inadequate discharge planning | Equipment | Medication dispensing | Communication with parents/ patients |  | 1 |
| Inadequate examination | Transfer of information | Transfer of information |  |  | 1 |
| Inadequate examination | Documentation | Transfer of information |  |  | 1 |
| Inadequate examination | Equipment |  |  |  | 1 |
| Inadequate triaging | Other administrative |  |  |  | 1 |
| Inadequate triaging | Transfer of information |  |  |  | 1 |
| Inadequate triaging | Documentation | Inadequate history | Communication with parents/ patients | Other | 1 |
| Inadequate triaging | Inadequate triaging |  |  |  | 1 |
| Inadequate triaging | Inadequate history | Transfer of information |  |  | 1 |
| Inadequate triaging | Inadequate history | Failure to identify at risk/ vulnerable child |  |  | 1 |
| Inadequate triaging | Inadequate history | Communication with parents/ patients | Other |  | 1 |
| Inadequate triaging | Inadequate history | Communication with parents/ patients | Documentation |  | 1 |
| Inadequate triaging | Inadequate history | Communication between professionals |  |  | 1 |
| Inadequate triaging | Medication dispensing |  |  |  | 1 |
| Inadequate triaging | Communication between professionals | Documentation |  |  | 1 |
| Inadequate triaging | Equipment |  |  |  | 1 |
| Incomplete referral | Appointment management |  |  |  | 1 |
| Incomplete referral | Insufficient assessment (non-specific) |  |  |  | 1 |
| Incomplete referral | Inadequate examination |  |  |  | 1 |
| Incomplete referral | Communication with parents/ patients | Inadequate history | Delayed assessment | Other | 1 |
| Incomplete referral | Incomplete referral |  |  |  | 1 |
| Incomplete referral | Incomplete referral | Treatment decision |  |  | 1 |
| Incomplete referral | Communication with parents/ patients |  |  |  | 1 |
| Insufficient assessment (non-specific) | Transfer of information |  |  |  | 1 |
| Insufficient assessment (non-specific) | Transfer of patients |  |  |  | 1 |
| Insufficient assessment (non-specific) | Other |  |  |  | 1 |
| Insufficient assessment (non-specific) | Documentation | Documentation |  |  | 1 |
| Insufficient assessment (non-specific) | Documentation | Failure to identify at risk/ vulnerable child |  |  | 1 |
| Insufficient assessment (non-specific) | Medication prescribing |  |  |  | 1 |
| Insufficient assessment (non-specific) | Investigations |  |  |  | 1 |
| Insufficient assessment (non-specific) | Communication with parents/ patients |  |  |  | 1 |
| Insufficient assessment (non-specific) | Communication between professionals |  |  |  | 1 |
| Investigations | Inadequate discharge planning |  |  |  | 1 |
| Investigations | Other administrative |  |  |  | 1 |
| Investigations | Transfer of information |  |  |  | 1 |
| Investigations | Investigations | Transfer of patients |  |  | 1 |
| Investigations | Communication with parents/ patients | Documentation |  |  | 1 |
| Investigations | Transfer of information | Transfer of information |  |  | 1 |
| Investigations | Documentation | Equipment |  |  | 1 |
| Medication administration | Inadequate discharge planning |  |  |  | 1 |
| Medication administration | Other medication |  |  |  | 1 |
| Medication administration | Medication administration |  |  |  | 1 |
| Medication administration | Medication monitoring | Appointment management | Transfer of information |  | 1 |
| Medication administration | Communication with parents/ patients | Medication dispensing | Medication prescribing |  | 1 |
| Medication administration | Communication with parents/ patients | Communication with parents/ patients |  |  | 1 |
| Medication administration | Communication with parents/ patients | Equipment |  |  | 1 |
| Medication administration | Equipment | Communication with parents/ patients |  |  | 1 |
| Medication administration | Documentation |  |  |  | 1 |
| Medication administration | Medication prescribing | Communication with parents/ patients |  |  | 1 |
| Medication administration | Medication dispensing | Medication dispensing |  |  | 1 |
| Medication administration | Documentation | Communication with parents/ patients |  |  | 1 |
| Medication administration | Access to care |  |  |  | 1 |
| Medication administration | Documentation | Transfer of information |  |  | 1 |
| Medication administration | Documentation |  |  |  | 1 |
| Medication administration | Other medication |  |  |  | 1 |
| Medication administration | Medication prescribing |  |  |  | 1 |
| Medication administration | Medication prescribing | Equipment | Communication with parents/ patients |  | 1 |
| Medication administration | Medication dispensing | Medication prescribing |  |  | 1 |
| Medication administration | Communication between professionals |  |  |  | 1 |
| Medication dispensing | Medication dispensing | Equipment |  |  | 1 |
| Medication dispensing | Documentation |  |  |  | 1 |
| Medication dispensing | Other medication |  |  |  | 1 |
| Medication dispensing | Medication prescribing | Medication prescribing |  |  | 1 |
| Medication dispensing | Equipment |  |  |  | 1 |
| Medication dispensing | Other administrative |  |  |  | 1 |
| Medication monitoring |  |  |  |  | 1 |
| Medication monitoring | Transfer of information | Transfer of information |  |  | 1 |
| Medication monitoring | Inadequate discharge planning | Transfer of information |  |  | 1 |
| Medication monitoring | Transfer of information |  |  |  | 1 |
| Medication monitoring | Insufficient assessment (non-specific | Documentation |  |  | 1 |
| Medication monitoring | Investigations |  |  |  | 1 |
| Medication prescribing | Transfer of information |  |  |  | 1 |
| Medication prescribing | Transfer of information | Inadequate discharge planning |  |  | 1 |
| Medication prescribing | Equipment |  |  |  | 1 |
| Medication prescribing | Inadequate history |  |  |  | 1 |
| Medication prescribing | Treatment decision | Communication between professionals |  |  | 1 |
| Medication prescribing | Medication prescribing |  |  |  | 1 |
| Medication prescribing | Medication monitoring | Access to care |  |  | 1 |
| Medication prescribing | Communication between professionals |  |  |  | 1 |
| Medication prescribing | Medication dispensing | Medication prescribing |  |  | 1 |
| Medication prescribing | Inadequate history | Documentation | Communication with parents/ patients |  | 1 |
| Medication prescribing | Communication with parents/ patients |  |  |  | 1 |
| Medication prescribing | Medication dispensing |  |  |  | 1 |
| Other | Communication with parents/ patients |  |  |  | 1 |
| Other | Communication with parents/ patients | Communication between professionals |  |  | 1 |
| Other | Communication between professionals |  |  |  | 1 |
| Other | Other administrative |  |  |  | 1 |
| Other | Access to care | Incomplete referral |  |  | 1 |
| Other | Other | Failure to refer when appropriate |  |  | 1 |
| Other | Other | Failure to refer when appropriate | Appointment management | Referral administrative issues | 1 |
| Other | Transfer of information | Other administrative |  |  | 1 |
| Other | Delayed referral | Documentation | Failure to identify at risk/ vulnerable child |  | 1 |
| Other | Delayed referral | Inadequate triaging | Inadequate history |  | 1 |
| Other | Failure to refer when appropriate |  |  |  | 1 |
| Other | Referral administrative issues |  |  |  | 1 |
| Other | Failure to identify at risk/ vulnerable child |  |  |  | 1 |
| Other | Inadequate discharge planning |  |  |  | 1 |
| Other | Treatment and procedures | Inadequate discharge planning | Failure to refer when appropriate |  | 1 |
| Other | Other medication | Other medication |  |  | 1 |
| Other administrative | Communication with parents/ patients |  |  |  | 1 |
| Other administrative | Appointment management |  |  |  | 1 |
| Other administrative | Communication with parents/ patients | Documentation |  |  | 1 |
| Other administrative | Communication between professionals |  |  |  | 1 |
| Other diagnosis and assessment | Delayed referral | Inadequate triaging | Failure to identify at risk/ vulnerable child | Inadequate examination | 1 |
| Other medication | Medication dispensing | Medication prescribing |  |  | 1 |
| Referral administrative issues | Other administrative |  |  |  | 1 |
| Referral administrative issues | Appointment management |  |  |  | 1 |
| Referral administrative issues | Transfer of information |  |  |  | 1 |
| Referral administrative issues | Communication with parents/ patients |  |  |  | 1 |
| Referral administrative issues | Insufficient assessment (non-specific) | Communication between professionals |  |  | 1 |
| Referral administrative issues | Inadequate triaging |  |  |  | 1 |
| Transfer of information | Inadequate discharge planning |  |  |  | 1 |
| Transfer of information | Inadequate discharge planning | Communication with parents/ patients |  |  | 1 |
| Transfer of information | Access to care | Documentation |  |  | 1 |
| Transfer of information | Referral administrative issues |  |  |  | 1 |
| Transfer of information | Inadequate discharge planning |  |  |  | 1 |
| Transfer of information | Communication between professionals | Transfer of information |  |  | 1 |
| Transfer of information | Inadequate triaging |  |  |  | 1 |
| Transfer of information | Inadequate history |  |  |  | 1 |
| Transfer of patients | Access to care | Appointment management |  |  | 1 |
| Transfer of patients | Transfer of information |  |  |  | 1 |
| Transfer of patients | Incomplete referral | Incomplete referral |  |  | 1 |
| Transfer of patients | Failure to identify at risk/ vulnerable child |  |  |  | 1 |
| Transfer of patients | Insufficient assessment (non-specific) | Communication between professionals |  |  | 1 |
| Transfer of patients | Delayed assessment |  |  |  | 1 |
| Transfer of patients | Communication with parents/ patients |  |  |  | 1 |
| Transfer of patients | Incomplete referral |  |  |  | 1 |
| Transfer of patients | Communication between professionals |  |  |  | 1 |
| Transfer of patients | Diagnosis | Failure to identify at risk/ vulnerable child | Investigations |  | 1 |
| Treatment and procedures | Treatment and procedures | Communication with parents/ patients |  |  | 1 |
| Treatment and procedures | Failure to refer when appropriate |  |  |  | 1 |
| Treatment and procedures | Diagnosis |  |  |  | 1 |
| Treatment and procedures | Insufficient assessment (non-specific) |  |  |  | 1 |
| Treatment and procedures | Other diagnosis and assessment | Failure to identify at risk/ vulnerable child |  |  | 1 |
| Treatment and procedures | Treatment and procedures | Treatment and procedures |  |  | 1 |
| Treatment and procedures | Medication dispensing | Medication dispensing |  |  | 1 |
| Treatment and procedures | Investigations | Equipment |  |  | 1 |
| Treatment and procedures | Inadequate history |  |  |  | 1 |
| Treatment and procedures | Appointment management | Treatment and procedures |  |  | 1 |
| Treatment and procedures | Access to care | Incomplete referral |  |  | 1 |
| Treatment and procedures | Transfer of information | Failure to refer when appropriate.4 | Inadequate discharge planning |  | 1 |
| Treatment and procedures | Transfer of patients |  |  |  | 1 |
| Treatment and procedures | Delayed referral |  |  |  | 1 |
| Treatment and procedures | Incomplete referral |  |  |  | 1 |
| Treatment and procedures | Incomplete referral | Incomplete referral |  |  | 1 |
| Treatment and procedures | Referral administrative issues | Appointment management |  |  | 1 |
| Treatment and procedures | Diagnosis | Investigations | Investigations |  | 1 |
| Treatment and procedures | Diagnosis | Investigations |  |  | 1 |
| Treatment and procedures | Inadequate triaging | Inadequate history |  |  | 1 |
| Treatment and procedures | Delayed assessment | Transfer of information |  |  | 1 |
| Treatment and procedures | Delayed assessment | Inadequate triaging | Communication between professionals |  | 1 |
| Treatment and procedures | Delayed assessment | Inadequate discharge planning | Incomplete referral |  | 1 |
| Treatment and procedures | Treatment and procedures | Appointment management |  |  | 1 |
| Treatment and procedures | Medication dispensing |  |  |  | 1 |
| Treatment and procedures | Investigations | Transfer of information | Inadequate discharge planning |  | 1 |
| Treatment and procedures | Investigations | Communication between professionals |  |  | 1 |
| Treatment and procedures | Investigations | Equipment |  |  | 1 |
| Treatment and procedures | Investigations |  |  |  | 1 |
| Treatment and procedures | Equipment | Other administrative |  |  | 1 |
| Treatment decision | Access to care | Access to care |  |  | 1 |
| Treatment decision | Other diagnosis and assessment |  |  |  | 1 |
| Treatment decision | Appointment management |  |  |  | 1 |
| Treatment decision | Transfer of information |  |  |  | 1 |
| Treatment decision | Diagnosis | Transfer of information | Transfer of information |  | 1 |
| Treatment decision | Insufficient assessment (non-specific) |  |  |  | 1 |
| Treatment decision | Inadequate triaging | Inadequate history | Documentation |  | 1 |
| Treatment decision | Inadequate examination |  |  |  | 1 |
| Treatment decision | Medication dispensing | Equipment |  |  | 1 |
| Treatment decision | Other medication | Transfer of information | Failure to identify at risk/ vulnerable child |  | 1 |
| Treatment decision | Other medication | Access to care | Documentation |  | 1 |
| Treatment decision | Equipment |  |  |  | 1 |
| Treatment decision | Delayed referral |  |  |  | 1 |
| Treatment decision | Transfer of information | Incomplete referral | Delayed referral | Inadequate discharge planning | 1 |
| Treatment decision | Insufficient assessment (non-specific |  |  |  | 1 |
| Treatment decision | Inadequate examination |  |  |  | 1 |
| Treatment decision | Failure to identify at risk/ vulnerable child | Diagnosis |  |  | 1 |
| Treatment decision | Inadequate discharge planning | Failure to identify at risk/ vulnerable child |  |  | 1 |
| Treatment decision | Other medication | Communication between professionals |  |  | 1 |
| Treatment decision | Investigations |  |  |  | 1 |
| Treatment decision | Investigations | Documentation |  |  | 1 |
| Treatment decision | Investigations | Transfer of information |  |  | 1 |
| Treatment decision | Communication between professionals | Communication with parents/ patients |  |  | 1 |
| Treatment decision | Equipment | Transfer of information |  |  | 1 |
| Treatment decision | Equipment | Investigations |  |  | 1 |
| Treatment decision | Equipment | Transfer of information | Inadequate discharge planning |  | 1 |
| **Total** |  |  |  |  | **1051** |
