# Supplementary material for: Patient Safety Incidents Involving Sick Children in Primary Care in England and Wales: A Mixed Methods Analysis
Source: PLoS Med. 2017 Jan 17;14(1):e1002217. doi: 10.1371/journal.pmed.1002217 (PMC5240916; doi:10.1371/journal.pmed.1002217)
Supplement: S4 Table — (DOCX) [file pmed.1002217.s005.docx]

| **S4 Table: the frequency of combinations of contributory factors for each primary incident type** | | | | | |
| --- | --- | --- | --- | --- | --- |
| **Primary incident** | **Contributory factors** | | | | **Frequency of combination** |
| Medication dispensing | Mistakes |  |  |  | 78 |
| Inadequate triaging | Failure to follow protocol |  |  |  | 67 |
| Transfer of patient information | Continuity of care |  |  |  | 27 |
| Communication with parents/ patients | Failure to follow protocol |  |  |  | 26 |
| Diagnostic issues |  |  |  |  | 26 |
| Medication dispensing | Mistake | Equipment factors |  |  | 26 |
| Inadequate triaging | Critical thinking |  |  |  | 22 |
| Delayed referral | Failure to follow protocol |  |  |  | 17 |
| Inadequate triaging | Failure to follow protocol | Critical thinking |  |  | 15 |
| Medication administering | Mistakes |  |  |  | 14 |
| Medication dispensing | Mistakes | Working conditions |  |  | 13 |
| Medication dispensing | Failure to follow protocol |  |  |  | 12 |
| Delayed assessment | Failure to follow protocol |  |  |  | 10 |
| Medication prescribing | Mistakes |  |  |  | 10 |
| Access to care | Continuity of care |  |  |  | 9 |
| Delayed assessment | Continuity of care |  |  |  | 9 |
| Documentation | Failure to follow protocol |  |  |  | 9 |
| Medication administering | Patient age |  |  |  | 8 |
| Medication dispensing | Patient age |  |  |  | 8 |
| Medication prescribing | Patient age |  |  |  | 8 |
| Treatment decisions | Working conditions |  |  |  | 8 |
| Delayed referral | Critical thinking |  |  |  | 7 |
| Failure to refer when appropriate | Failure to follow protocol |  |  |  | 7 |
| Medication dispensing | Equipment factors |  |  |  | 7 |
| Medication dispensing | Inadequate guidelines |  |  |  | 7 |
| Medication dispensing | Mistakes | Failure to follow protocol |  |  | 7 |
| Other | Failure to follow protocol |  |  |  | 7 |
| Access to care | Failure to follow protocol |  |  |  | 6 |
| Communication with parents/ patients | Mistakes |  |  |  | 6 |
| Delayed assessment | Service availability |  |  |  | 6 |
| Inadequate history taking |  |  |  |  | 6 |
| Inadequate history taking | Failure to follow protocol |  |  |  | 6 |
| Inadequate triaging | Patient/ parent behaviour |  |  |  | 6 |
| Medication administering | Failure to follow protocol |  |  |  | 6 |
| Medication administering | Equipment factors | Working conditions |  |  | 6 |
| Medication dispensing | Mistakes | Working conditions | Working conditions |  | 6 |
| Transfer of patient information | Failure to follow protocol |  |  |  | 6 |
| Treatment and procedures | Patient/ parent behaviour |  |  |  | 6 |
| Treatment and procedures | Continuity of care |  |  |  | 6 |
| Communication between professionals |  |  |  |  | 5 |
| Delayed referral | Patient/ parent behaviour |  |  |  | 5 |
| Delayed referral | Failure to follow protocol | Critical thinking |  |  | 5 |
| Diagnostic issues | Failure to follow protocol |  |  |  | 5 |
| Other | Patient health |  |  |  | 5 |
| Other | Patient/ parent behaviour |  |  |  | 5 |
| Transfer of patient information | Continuity of care | Patient/ parent geography |  |  | 5 |
| Treatment decisions | Inadequate guidelines |  |  |  | 5 |
| Access to care | Working conditions |  |  |  | 4 |
| Communication between professionals | Failure to follow protocol |  |  |  | 4 |
| Communication with parents/ patients | Patient age |  |  |  | 4 |
| Communication with parents/ patients | Staff knowledge |  |  |  | 4 |
| Communication with parents/ patients | Failure to follow protocol | Staff knowledge |  |  | 4 |
| Delayed assessment | Inadequate guidelines |  |  |  | 4 |
| Delayed assessment | Working conditions |  |  |  | 4 |
| Delayed referral | Staff knowledge |  |  |  | 4 |
| Inadequate triaging | Mistakes |  |  |  | 4 |
| Inadequate triaging | Education and training |  |  |  | 4 |
| Medication administering | Patient/ parent behaviour |  |  |  | 4 |
| Medication administering | Patient/ parent knowledge |  |  |  | 4 |
| Medication dispensing | Working conditions |  |  |  | 4 |
| Medication dispensing | Mistake | Equipment factors | Working conditions |  | 4 |
| Medication prescribing | Failure to follow protocol | Patient age |  |  | 4 |
| Treatment and procedures | Patient age |  |  |  | 4 |
| Treatment decisions | Failure to follow protocol |  |  |  | 4 |
| Treatment decisions | Continuity of care |  |  |  | 4 |
| Access to care | Patient health |  |  |  | 3 |
| Access to care | Service availability |  |  |  | 3 |
| Access to care | Patient/ parent geography | Continuity of care |  |  | 3 |
| Communication between professionals | Continuity of care |  |  |  | 3 |
| Communication between professionals | Working conditions |  |  |  | 3 |
| Communication with parents/ patients | Patient health |  |  |  | 3 |
| Communication with parents/ patients | Patient/ parent knowledge |  |  |  | 3 |
| Communication with parents/ patients | Inadequate guidelines |  |  |  | 3 |
| Communication with parents/ patients | Continuity of care |  |  |  | 3 |
| Communication with parents/ patients | Failure to follow protocol | Patient age |  |  | 3 |
| Communication with parents/ patients | Failure to follow protocol | Critical thinking |  |  | 3 |
| Delayed referral | Continuity of care |  |  |  | 3 |
| Delayed referral | Working conditions |  |  |  | 3 |
| Delayed referral | Failure to follow protocol | Staff knowledge |  |  | 3 |
| Diagnostic issues | Patient health |  |  |  | 3 |
| Diagnostic issues | Staff knowledge | Education and training |  |  | 3 |
| Documentation | Mistakes |  |  |  | 3 |
| Equipment | Inadequate guidelines |  |  |  | 3 |
| Equipment | Education and training |  |  |  | 3 |
| Equipment | Working conditions |  |  |  | 3 |
| Inadequate triaging | Staff knowledge |  |  |  | 3 |
| Inadequate triaging | Failure to follow protocol | Patient age |  |  | 3 |
| Incorrect/ incomplete referral | Failure to follow protocol |  |  |  | 3 |
| Insufficient assessment (non-specific) | Patient age |  |  |  | 3 |
| Medication dispensing | Staff knowledge |  |  |  | 3 |
| Medication dispensing | Mistakes | Patient age |  |  | 3 |
| Medication dispensing | Mistake | Inadequate guidelines |  |  | 3 |
| Medication dispensing | Working conditions | Working conditions |  |  | 3 |
| Medication dispensing | Mistakes | Working conditions | Equipment |  | 3 |
| Medication dispensing | Mistake | Equipment factors | Working conditions | Working conditions | 3 |
| Medication prescribing | Staff knowledge |  |  |  | 3 |
| Other administrative |  |  |  |  | 3 |
| Other medication | Continuity of care |  |  |  | 3 |
| Transfer of patient information | Patient/ parent geography |  |  |  | 3 |
| Treatment and procedures | Patient health |  |  |  | 3 |
| Treatment and procedures | Equipment factors |  |  |  | 3 |
| Treatment and procedures | Working conditions |  |  |  | 3 |
| Communication between professionals | Mistake |  |  |  | 2 |
| Communication with parents/ patients | Critical thinking |  |  |  | 2 |
| Communication with parents/ patients | Education and training |  |  |  | 2 |
| Communication with parents/ patients | Working conditions |  |  |  | 2 |
| Communication with parents/ patients | Failure to follow protocol | Patient/ parent geography |  |  | 2 |
| Delayed assessment | Patient/ parent geography |  |  |  | 2 |
| Delayed assessment | Failure to follow protocol | Staff knowledge |  |  | 2 |
| Delayed assessment | Service availability | Inadequate guidelines |  |  | 2 |
| Delayed assessment | Service availability | Working conditions |  |  | 2 |
| Delayed assessment | Service availability | Working conditions |  |  | 2 |
| Delayed referral | Mistakes |  |  |  | 2 |
| Delayed referral | Inadequate guidelines |  |  |  | 2 |
| Documentation | Staff knowledge |  |  |  | 2 |
| Equipment | Non-specific organisational issues |  |  |  | 2 |
| Failure to refer when appropriate | Patient age |  |  |  | 2 |
| Failure to refer when appropriate | Staff knowledge |  |  |  | 2 |
| Inadequate discharge planning | Patient/ parent behaviour |  |  |  | 2 |
| Inadequate discharge planning | Failure to follow protocol |  |  |  | 2 |
| Inadequate history taking | Critical thinking |  |  |  | 2 |
| Inadequate triaging | Patient age |  |  |  | 2 |
| Inadequate triaging | Inadequate guidelines |  |  |  | 2 |
| Inadequate triaging | Failure to follow protocol | Patient/ parent health |  |  | 2 |
| Inadequate triaging | Critical thinking | Patient age |  |  | 2 |
| Inadequate triaging | Failure to follow protocol | Staff knowledge |  |  | 2 |
| Inadequate triaging | Patient health | Critical thinking |  |  | 2 |
| Inadequate triaging | Failure to follow protocol | Education and training |  |  | 2 |
| Incorrect/ incomplete referral | Patient age |  |  |  | 2 |
| Incorrect/ incomplete referral | Inadequate guidelines |  |  |  | 2 |
| Incorrect/ incomplete referral | Education and training |  |  |  | 2 |
| Incorrect/ incomplete referral | Failure to follow protocol | Education and training |  |  | 2 |
| Insufficient assessment (non-specific) | Failure to follow protocol |  |  |  | 2 |
| Insufficient assessment (non-specific) | Working conditions |  |  |  | 2 |
| Insufficient assessment (non-specific) | Patient age | Inadequate guidelines |  |  | 2 |
| Investigations | Inadequate guidelines |  |  |  | 2 |
| Medication administering | Equipment factors |  |  |  | 2 |
| Medication administering | Education and training |  |  |  | 2 |
| Medication administering | Continuity of care |  |  |  | 2 |
| Medication administering | Working conditions |  |  |  | 2 |
| Medication administering | Failure to follow protocol | Patient/ parent knowledge |  |  | 2 |
| Medication dispensing | Education and training |  |  |  | 2 |
| Medication dispensing | Mistake | Staff knowledge |  |  | 2 |
| Medication dispensing | Mistake | Mistake |  |  | 2 |
| Medication dispensing | Patient age | Mistake |  |  | 2 |
| Medication dispensing | Mistake | Other staff factors |  |  | 2 |
| Medication dispensing | Patient age | Working conditions |  |  | 2 |
| Medication dispensing | Staff knowledge | Education and training |  |  | 2 |
| Medication dispensing | Mistakes | Equipment factors | Failure to follow protocol |  | 2 |
| Medication dispensing | Mistake | Staff knowledge | Inadequate guidelines |  | 2 |
| Medication prescribing | Inadequate guidelines |  |  |  | 2 |
| Medication prescribing | Patient age | Failure to follow protocol |  |  | 2 |
| Medication prescribing | Mistakes | Equipment factors |  |  | 2 |
| Other medication | Inadequate guidelines |  |  |  | 2 |
| Transfer of patient information | Patient/ parent behaviour |  |  |  | 2 |
| Transport/ transfer of patients | Patient/ parent geography |  |  |  | 2 |
| Transport/ transfer of patients | Failure to follow protocol |  |  |  | 2 |
| Transport/ transfer of patients | Service availability |  |  |  | 2 |
| Transport/ transfer of patients | Working conditions | Service availability |  |  | 2 |
| Treatment and procedures | Mistakes |  |  |  | 2 |
| Treatment and procedures | Staff knowledge |  |  |  | 2 |
| Treatment and procedures | Inadequate guidelines |  |  |  | 2 |
| Treatment and procedures | Education and training |  |  |  | 2 |
| Treatment and procedures | Service availability |  |  |  | 2 |
| Treatment and procedures | Mistakes | Patient/ parent behaviour |  |  | 2 |
| Treatment and procedures | Patient/ parent behaviour | Patient/ parent health |  |  | 2 |
| Treatment and procedures | Working conditions | Continuity of care |  |  | 2 |
| Treatment decisions | Equipment factors |  |  |  | 2 |
| Treatment decisions | Continuity of care | Inadequate guidelines |  |  | 2 |
| Access to care | Patient/ parent geography |  |  |  | 1 |
| Access to care | Patient/ parent knowledge |  |  |  | 1 |
| Access to care | Patient/ parent behaviour |  |  |  | 1 |
| Access to care | Critical thinking |  |  |  | 1 |
| Access to care | Education and training |  |  |  | 1 |
| Access to care | Continuity of care | Patient age |  |  | 1 |
| Access to care | Mistake | Failure to follow protocol |  |  | 1 |
| Access to care | Failure to follow protocol | Staff knowledge |  |  | 1 |
| Access to care | Patient health | Failure to follow protocol |  |  | 1 |
| Access to care | Service availability | Continuity of care |  |  | 1 |
| Access to care | Failure to follow protocol | Service availability |  |  | 1 |
| Access to care | Looked-after child | Patient/ parent geography | Inadequate guidelines |  | 1 |
| Appointment management | Patient/ parent geography |  |  |  | 1 |
| Appointment management | Patient age |  |  |  | 1 |
| Appointment management | Mistake |  |  |  | 1 |
| Appointment management | Service availability |  |  |  | 1 |
| Appointment management | Working conditions |  |  |  | 1 |
| Appointment management | Mistakes | Failure to follow protocol |  |  | 1 |
| Communication between professionals | Continuity of care | Patient/ parent behaviour |  |  | 1 |
| Communication between professionals | Failure to follow protocol | Inadequate guidelines |  |  | 1 |
| Communication between professionals | Working conditions | Continuity of care |  |  | 1 |
| Communication between professionals | Critical thinking | Failure to follow protocol | Education and training |  | 1 |
| Communication with parents/ patients | Equipment factors |  |  |  | 1 |
| Communication with parents/ patients | Service availability |  |  |  | 1 |
| Communication with parents/ patients | Patient/ parent knowledge | Patient/ parent knowledge |  |  | 1 |
| Communication with parents/ patients | Failure to follow protocol | Patient/ parent health |  |  | 1 |
| Communication with parents/ patients | Mistake | Patient age |  |  | 1 |
| Communication with parents/ patients | Failure to follow protocol | Working conditions |  |  | 1 |
| Communication with parents/ patients | Failure to follow protocol | Education and training |  |  | 1 |
| Communication with parents/ patients | Failure to follow protocol | Staff knowledge | Patient health |  | 1 |
| Communication with parents/ patients | Patient /parent language | Service availability | Working conditions |  | 1 |
| Communication with parents/ patients | Failure to follow protocol | Patient age | Education and training |  | 1 |
| Communication with parents/ patients | Failure to follow protocol | Critical thinking | Service availability |  | 1 |
| Communication with parents/ patients | Patient health | Staff knowledge | Education and training | Failure to follow protocol | 1 |
| Delayed assessment | Looked-after child |  |  |  | 1 |
| Delayed assessment | Staff knowledge |  |  |  | 1 |
| Delayed assessment | Continuity of care | Patient/ parent geography |  |  | 1 |
| Delayed assessment | Service availability | Patient/ parent knowledge |  |  | 1 |
| Delayed assessment | Continuity of care | Patient/ parent health |  |  | 1 |
| Delayed assessment | Continuity of care | Patient age |  |  | 1 |
| Delayed assessment | Continuity of care | Failure to follow protocol |  |  | 1 |
| Delayed assessment | Service availability | Failure to follow protocol |  |  | 1 |
| Delayed assessment | Education and training | Failure to follow protocol |  |  | 1 |
| Delayed assessment | Patient age | Critical thinking |  |  | 1 |
| Delayed assessment | Continuity of care | Critical thinking |  |  | 1 |
| Delayed assessment | Patient /parent language | Service availability |  |  | 1 |
| Delayed assessment | Patient/ parent geography | Continuity of care |  |  | 1 |
| Delayed assessment | Patient health | Continuity of care |  |  | 1 |
| Delayed assessment | Continuity of care | Working conditions |  |  | 1 |
| Delayed assessment | Failure to follow protocol | Working conditions |  |  | 1 |
| Delayed assessment | Working conditions | Working conditions |  |  | 1 |
| Delayed assessment | Critical thinking | Education and training |  |  | 1 |
| Delayed assessment | Patient/ parent behaviour | Service availability |  |  | 1 |
| Delayed assessment | Failure to follow protocol | Staff knowledge | Education and training |  | 1 |
| Delayed referral | Patient /parent language |  |  |  | 1 |
| Delayed referral | Looked-after child |  |  |  | 1 |
| Delayed referral | Education and training |  |  |  | 1 |
| Delayed referral | Service availability |  |  |  | 1 |
| Delayed referral | Patient/ parent ethnicity | Inadequate guidelines |  |  | 1 |
| Delayed referral | Failure to follow protocol | Inadequate guidelines |  |  | 1 |
| Delayed referral | Mistakes | Inadequate guidelines |  |  | 1 |
| Delayed referral | Patient /parent language | Service availability |  |  | 1 |
| Delayed referral | Inadequate guidelines | Continuity of care |  |  | 1 |
| Delayed referral | Working conditions | Working conditions |  |  | 1 |
| Delayed referral | Failure to follow protocol | Education and training |  |  | 1 |
| Delayed referral | Critical thinking | Patient/ parent knowledge | Patient/ parent geography |  | 1 |
| Delayed referral | Critical thinking | Education and training | Patient age |  | 1 |
| Delayed referral | Failure to follow protocol | Critical thinking | Staff knowledge |  | 1 |
| Delayed referral | Patient age | Failure to follow protocol | Inadequate guidelines |  | 1 |
| Delayed referral | Staff knowledge | Failure to follow protocol | Working conditions |  | 1 |
| Delayed referral | Failure to follow protocol | Staff knowledge | Education and training |  | 1 |
| Delayed referral | Service availability | Working conditions | Working conditions | Continuity of care | 1 |
| Diagnostic issues | Patient/ parent behaviour |  |  |  | 1 |
| Diagnostic issues | Patient age |  |  |  | 1 |
| Diagnostic issues | Staff knowledge |  |  |  | 1 |
| Diagnostic issues | Inadequate guidelines |  |  |  | 1 |
| Diagnostic issues | Patient health | Staff knowledge |  |  | 1 |
| Diagnostic issues | Failure to follow protocol | Staff knowledge |  |  | 1 |
| Diagnostic issues | Inadequate guidelines | Staff knowledge |  |  | 1 |
| Diagnostic issues | Patient/ parent knowledge | Working conditions |  |  | 1 |
| Documentation | Patient/ parent knowledge |  |  |  | 1 |
| Documentation | Critical thinking |  |  |  | 1 |
| Documentation | Continuity of care |  |  |  | 1 |
| Documentation | Service availability |  |  |  | 1 |
| Documentation | Failure to follow protocol | Staff knowledge |  |  | 1 |
| Documentation | Failure to follow protocol | Critical thinking |  |  | 1 |
| Documentation | Failure to follow protocol | Mistake |  |  | 1 |
| Documentation | Service availability | Continuity of care |  |  | 1 |
| Documentation | Mistakes | Continuity of care |  |  | 1 |
| Equipment | Patient/ parent geography |  |  |  | 1 |
| Equipment | Patient/ parent knowledge |  |  |  | 1 |
| Equipment | Patient health |  |  |  | 1 |
| Equipment | Mistakes |  |  |  | 1 |
| Equipment | Equipment factors |  |  |  | 1 |
| Equipment | Continuity of care |  |  |  | 1 |
| Equipment | Environmental |  |  |  | 1 |
| Equipment | Failure to follow protocol | Inadequate guidelines |  |  | 1 |
| Equipment | Continuity of care | Inadequate guidelines |  |  | 1 |
| Equipment | Patient age | Continuity of care |  |  | 1 |
| Equipment | Continuity of care | Working conditions |  |  | 1 |
| Equipment | Staff knowledge | Education and training |  |  | 1 |
| Equipment | Staff knowledge | Inadequate guidelines | Education and training |  | 1 |
| Failure to arrange follow up | Mistake | Continuity of care |  |  | 1 |
| Failure to identify at risk child | Critical thinking |  |  |  | 1 |
| Failure to identify at risk child | Failure to follow protocol | Critical thinking |  |  | 1 |
| Failure to identify at risk child | Inadequate guidelines | Education and training |  |  | 1 |
| Failure to identify at risk child | Failure to follow protocol | Staff knowledge | Education and training |  | 1 |
| Failure to refer when appropriate | Patient /parent language |  |  |  | 1 |
| Failure to refer when appropriate | Critical thinking |  |  |  | 1 |
| Failure to refer when appropriate | Education and training | Patient/ parent behaviour |  |  | 1 |
| Failure to refer when appropriate | Critical thinking | Patient/ parent behaviour |  |  | 1 |
| Failure to refer when appropriate | Failure to follow protocol | Patient age |  |  | 1 |
| Failure to refer when appropriate | Failure to follow protocol | Critical thinking |  |  | 1 |
| Failure to refer when appropriate | Continuity of care | Critical thinking |  |  | 1 |
| Failure to refer when appropriate | Failure to follow protocol | Mistake |  |  | 1 |
| Failure to refer when appropriate | Staff knowledge | Inadequate guidelines |  |  | 1 |
| Failure to refer when appropriate | Failure to follow protocol | Continuity of care |  |  | 1 |
| Failure to refer when appropriate | Service availability | Failure to follow protocol | Patient health |  | 1 |
| Failure to refer when appropriate | Critical thinking | Failure to follow protocol | Staff knowledge |  | 1 |
| Inadequate discharge planning | Continuity of care |  |  |  | 1 |
| Inadequate discharge planning | Service availability |  |  |  | 1 |
| Inadequate discharge planning | Working conditions |  |  |  | 1 |
| Inadequate discharge planning | Continuity of care | Patient/ parent health |  |  | 1 |
| Inadequate discharge planning | Patient age | Inadequate guidelines |  |  | 1 |
| Inadequate discharge planning | Continuity of care | Inadequate guidelines |  |  | 1 |
| Inadequate discharge planning | Staff knowledge | Education and training |  |  | 1 |
| Inadequate discharge planning | Staff knowledge | Education and training | Working conditions |  | 1 |
| Inadequate examination | Patient/ parent geography |  |  |  | 1 |
| Inadequate examination | Continuity of care |  |  |  | 1 |
| Inadequate examination | Patient age | Failure to follow protocol | Staff knowledge |  | 1 |
| Inadequate history taking | Patient/ parent geography |  |  |  | 1 |
| Inadequate history taking | Service availability |  |  |  | 1 |
| Inadequate history taking | Environmental |  |  |  | 1 |
| Inadequate history taking | Patient age | Patient/ parent health |  |  | 1 |
| Inadequate history taking | Staff knowledge | Education and training |  |  | 1 |
| Inadequate history taking | Failure to follow protocol | Working conditions | Patient/ parent behaviour |  | 1 |
| Inadequate triaging | Patient health |  |  |  | 1 |
| Inadequate triaging | Patient/ parent knowledge |  |  |  | 1 |
| Inadequate triaging | Equipment factors |  |  |  | 1 |
| Inadequate triaging | Patient/ parent behaviour | Patient/ parent knowledge |  |  | 1 |
| Inadequate triaging | Failure to follow protocol | Patient/ parent behaviour |  |  | 1 |
| Inadequate triaging | Critical thinking | Patient/ parent health |  |  | 1 |
| Inadequate triaging | Critical thinking | Other staff factors |  |  | 1 |
| Inadequate triaging | Patient /parent language | Failure to follow protocol |  |  | 1 |
| Inadequate triaging | Patient health | Failure to follow protocol |  |  | 1 |
| Inadequate triaging | Failure to follow protocol | Failure to follow protocol |  |  | 1 |
| Inadequate triaging | Patient age | Failure to follow protocol |  |  | 1 |
| Inadequate triaging | Mistake | Failure to follow protocol |  |  | 1 |
| Inadequate triaging | Critical thinking | Staff knowledge |  |  | 1 |
| Inadequate triaging | Patient/ parent behaviour | Critical thinking |  |  | 1 |
| Inadequate triaging | Mistake | Critical thinking |  |  | 1 |
| Inadequate triaging | Inadequate guidelines | Critical thinking |  |  | 1 |
| Inadequate triaging | Education and training | Critical thinking |  |  | 1 |
| Inadequate triaging | Service availability | Critical thinking |  |  | 1 |
| Inadequate triaging | Failure to follow protocol | Mistake |  |  | 1 |
| Inadequate triaging | Patient age | Inadequate guidelines |  |  | 1 |
| Inadequate triaging | Critical thinking | Inadequate guidelines |  |  | 1 |
| Inadequate triaging | Failure to follow protocol | Inadequate guidelines |  |  | 1 |
| Inadequate triaging | Inadequate guidelines | Continuity of care |  |  | 1 |
| Inadequate triaging | Continuity of care | Working conditions |  |  | 1 |
| Inadequate triaging | Failure to follow protocol | Service availability |  |  | 1 |
| Inadequate triaging | Inadequate guidelines | Service availability |  |  | 1 |
| Inadequate triaging | Failure to follow protocol | Critical thinking | Patient/ parent language |  | 1 |
| Inadequate triaging | Failure to follow protocol | Critical thinking | Patient age |  | 1 |
| Inadequate triaging | Patient/ parent knowledge | Inadequate guidelines | Critical thinking |  | 1 |
| Inadequate triaging | Inadequate guidelines | Education and training | Critical thinking |  | 1 |
| Inadequate triaging | Failure to follow protocol | Patient age | Critical thinking | Education and training | 1 |
| Incorrect/ incomplete referral | Patient health |  |  |  | 1 |
| Incorrect/ incomplete referral | Critical thinking |  |  |  | 1 |
| Incorrect/ incomplete referral | Staff knowledge |  |  |  | 1 |
| Incorrect/ incomplete referral | Working conditions |  |  |  | 1 |
| Incorrect/ incomplete referral | Mistakes | Patient age |  |  | 1 |
| Incorrect/ incomplete referral | Continuity of care | Failure to follow protocol |  |  | 1 |
| Incorrect/ incomplete referral | Patient age | Mistake |  |  | 1 |
| Incorrect/ incomplete referral | Inadequate guidelines | Education and training |  |  | 1 |
| Incorrect/ incomplete referral | Patient/ parent behaviour | Patient/ parent health | Failure to follow protocol |  | 1 |
| Insufficient assessment (non-specific) | Critical thinking |  |  |  | 1 |
| Insufficient assessment (non-specific) | Mistake |  |  |  | 1 |
| Insufficient assessment (non-specific) | Continuity of care |  |  |  | 1 |
| Insufficient assessment (non-specific) | Education and training |  |  |  | 1 |
| Insufficient assessment (non-specific) | Continuity of care | Patient/ parent geography |  |  | 1 |
| Insufficient assessment (non-specific) | Failure to follow protocol | Patient age |  |  | 1 |
| Insufficient assessment (non-specific) | Working conditions | Working conditions |  |  | 1 |
| Insufficient assessment (non-specific) | Failure to follow protocol | Patient age | Patient/ parent knowledge |  | 1 |
| Investigations | Patient health |  |  |  | 1 |
| Investigations | Patient/ parent behaviour |  |  |  | 1 |
| Investigations | Failure to follow protocol |  |  |  | 1 |
| Investigations | Mistakes |  |  |  | 1 |
| Investigations | Equipment factors |  |  |  | 1 |
| Investigations | Education and training |  |  |  | 1 |
| Investigations | Working conditions |  |  |  | 1 |
| Investigations | Mistake | Patient/ parent knowledge | Patient/ parent geography |  | 1 |
| Medication administering | Inadequate guidelines |  |  |  | 1 |
| Medication administering | Continuity of care | Patient/ parent geography |  |  | 1 |
| Medication administering | Inadequate guidelines | Patient/ parent behaviour |  |  | 1 |
| Medication administering | Patient/ parent behaviour | Patient/ parent knowledge |  |  | 1 |
| Medication administering | Patient age | Patient/ parent knowledge |  |  | 1 |
| Medication administering | Patient/ parent behaviour | Patient/ parent knowledge |  |  | 1 |
| Medication administering | Mistakes | Patient age |  |  | 1 |
| Medication administering | Staff knowledge | Patient age |  |  | 1 |
| Medication administering | Patient/ parent knowledge | Patient age |  |  | 1 |
| Medication administering | Mistake | Failure to follow protocol |  |  | 1 |
| Medication administering | Mistake | Failure to follow protocol |  |  | 1 |
| Medication administering | Patient age | Mistake |  |  | 1 |
| Medication administering | Failure to follow protocol | Failure to follow protocol |  |  | 1 |
| Medication administering | Mistakes | Equipment factors |  |  | 1 |
| Medication administering | Mistake | Equipment factors |  |  | 1 |
| Medication administering | Inadequate guidelines | Continuity of care |  |  | 1 |
| Medication administering | Mistake | Working conditions |  |  | 1 |
| Medication administering | Mistakes | Working conditions |  |  | 1 |
| Medication administering | Mistake | Working conditions |  |  | 1 |
| Medication administering | Mistake | Working conditions |  |  | 1 |
| Medication administering | Working conditions | Working conditions |  |  | 1 |
| Medication administering | Inadequate guidelines | Education and training |  |  | 1 |
| Medication administering | Patient/ parent knowledge | Education and training |  |  | 1 |
| Medication administering | Patient age | Mistake | Staff knowledge |  | 1 |
| Medication administering | Patient health | Patient/ parent knowledge | Continuity of care |  | 1 |
| Medication administering | Mistake | Failure to follow protocol | Continuity of care |  | 1 |
| Medication administering | Failure to follow protocol | Working conditions | Working conditions |  | 1 |
| Medication administering | Mistake | Working conditions | Working conditions |  | 1 |
| Medication administering | Mistakes | Failure to follow protocol | Working conditions |  | 1 |
| Medication administering | Patient age | Working conditions | Working conditions |  | 1 |
| Medication administering | Mistake | Working conditions | Education and training |  | 1 |
| Medication administering | Mistake | Patient/ parent health | Failure to follow protocol | Equipment | 1 |
| Medication administering | Continuity of care | Patient/ parent geography | Patient/ parent knowledge | Inadequate protocols | 1 |
| Medication dispensing | Patient health |  |  |  | 1 |
| Medication dispensing | Patient/ parent behaviour |  |  |  | 1 |
| Medication dispensing | Failure to follow protocol | Patient/ parent health |  |  | 1 |
| Medication dispensing | Patient age | Failure to follow protocol |  |  | 1 |
| Medication dispensing | Working conditions | Staff knowledge |  |  | 1 |
| Medication dispensing | Equipment factors | Mistake |  |  | 1 |
| Medication dispensing | Staff knowledge | Equipment factors |  |  | 1 |
| Medication dispensing | Mistake | Service availability |  |  | 1 |
| Medication dispensing | Working conditions | Continuity of care |  |  | 1 |
| Medication dispensing | Mistakes | Working conditions |  |  | 1 |
| Medication dispensing | Staff knowledge | Working conditions |  |  | 1 |
| Medication dispensing | Failure to follow protocol | Education and training |  |  | 1 |
| Medication dispensing | Mistake | Education and training |  |  | 1 |
| Medication dispensing | Inadequate guidelines | Education and training |  |  | 1 |
| Medication dispensing | Patient age | Failure to follow protocol | Mistake |  | 1 |
| Medication dispensing | Mistake | Equipment factors | Mistake |  | 1 |
| Medication dispensing | Mistakes | Working conditions | Other staff factors |  | 1 |
| Medication dispensing | Mistake | Patient age | Equipment |  | 1 |
| Medication dispensing | Patient health | Mistake | Equipment |  | 1 |
| Medication dispensing | Mistakes | Patient age | Inadequate guidelines |  | 1 |
| Medication dispensing | Equipment factors | Failure to follow protocol | Inadequate guidelines |  | 1 |
| Medication dispensing | Failure to follow protocol | Staff knowledge | Inadequate guidelines |  | 1 |
| Medication dispensing | Mistake | Education and training | Working conditions |  | 1 |
| Medication dispensing | Patient age | Failure to follow protocol | Working conditions |  | 1 |
| Medication dispensing | Mistake | Failure to follow protocol | Working conditions |  | 1 |
| Medication dispensing | Mistake | Staff knowledge | Working conditions |  | 1 |
| Medication dispensing | Mistake | Working conditions | Working conditions |  | 1 |
| Medication dispensing | Mistake | Mistake | Working conditions |  | 1 |
| Medication dispensing | Patient age | Staff knowledge | Education and training |  | 1 |
| Medication dispensing | Failure to follow protocol | Equipment factors | Education and training |  | 1 |
| Medication dispensing | Inadequate guidelines | Continuity of care | Patient/ parent knowledge | Patient/ parent behaviour | 1 |
| Medication dispensing | Failure to follow protocol | Working conditions | Staff knowledge | Patient age | 1 |
| Medication dispensing | Mistake | Mistake | Working conditions | Working conditions | 1 |
| Medication dispensing | Mistakes | Patient age | Working conditions | Working conditions | 1 |
| Medication prescribing | Patient/ parent geography |  |  |  | 1 |
| Medication prescribing | Patient /parent language |  |  |  | 1 |
| Medication prescribing | Patient/ parent behaviour |  |  |  | 1 |
| Medication prescribing | Patient health |  |  |  | 1 |
| Medication prescribing | Failure to follow protocol |  |  |  | 1 |
| Medication prescribing | Patient/ parent geography | Patient/ parent knowledge |  |  | 1 |
| Medication prescribing | Patient/ parent behaviour | Patient/ parent knowledge |  |  | 1 |
| Medication prescribing | Mistake | Patient age |  |  | 1 |
| Medication prescribing | Patient/ parent behaviour | Failure to follow protocol |  |  | 1 |
| Medication prescribing | Mistake | Failure to follow protocol |  |  | 1 |
| Medication prescribing | Mistake | Staff knowledge |  |  | 1 |
| Medication prescribing | Patient health | Mistake |  |  | 1 |
| Medication prescribing | Patient age | Equipment factors |  |  | 1 |
| Medication prescribing | Failure to follow protocol | Inadequate guidelines |  |  | 1 |
| Medication prescribing | Continuity of care | Inadequate guidelines |  |  | 1 |
| Medication prescribing | Staff knowledge | Failure to follow protocol | Patient age |  | 1 |
| Medication prescribing | Mistakes | Staff knowledge | Patient age |  | 1 |
| Medication prescribing | Mistake | Staff knowledge | Patient age |  | 1 |
| Medication prescribing | Continuity of care | Inadequate guidelines | Patient age |  | 1 |
| Other | Patient/ parent geography |  |  |  | 1 |
| Other | Looked-after child |  |  |  | 1 |
| Other | Inadequate guidelines |  |  |  | 1 |
| Other | Continuity of care |  |  |  | 1 |
| Other | Working conditions |  |  |  | 1 |
| Other | Continuity of care | Patient/ parent geography |  |  | 1 |
| Other | Continuity of care | Patient/ parent health |  |  | 1 |
| Other | Inadequate guidelines | Looked-after child |  |  | 1 |
| Other | Continuity of care | Patient age |  |  | 1 |
| Other | Continuity of care | Failure to follow protocol |  |  | 1 |
| Other | Continuity of care | Inadequate guidelines |  |  | 1 |
| Other | Staff knowledge | Working conditions |  |  | 1 |
| Other | Failure to follow protocol | Staff knowledge | Education and training |  | 1 |
| Other administrative | Patient age |  |  |  | 1 |
| Other administrative | Failure to follow protocol |  |  |  | 1 |
| Other administrative | Mistake |  |  |  | 1 |
| Other administrative | Inadequate guidelines |  |  |  | 1 |
| Other diagnosis and assessment | Patient age | Staff knowledge |  |  | 1 |
| Other medication | Patient/ parent geography |  |  |  | 1 |
| Other medication | Patient/ parent behaviour |  |  |  | 1 |
| Other medication | Mistakes |  |  |  | 1 |
| Other medication | Education and training |  |  |  | 1 |
| Other medication | Service availability | Patient/ parent behaviour |  |  | 1 |
| Other medication | Mistake | Staff knowledge |  |  | 1 |
| Other medication | Mistakes | Staff knowledge | Other staff factors |  | 1 |
| Other medication | Mistake | Staff knowledge | Education and training |  | 1 |
| Referral administrative issues | Mistake |  |  |  | 1 |
| Referral administrative issues | Service availability |  |  |  | 1 |
| Referral administrative issues | Failure to follow protocol | Patient/ parent health |  |  | 1 |
| Referral administrative issues | Continuity of care | Patient/ parent knowledge |  |  | 1 |
| Referral administrative issues | Continuity of care | Inadequate guidelines |  |  | 1 |
| Referral administrative issues | Education and training | Working conditions |  |  | 1 |
| Referral administrative issues | Looked-after child | Patient/ parent geography | Continuity of care |  | 1 |
| Transfer of patient information | Patient /parent language |  |  |  | 1 |
| Transfer of patient information | Mistakes |  |  |  | 1 |
| Transfer of patient information | Working conditions |  |  |  | 1 |
| Transfer of patient information | Failure to follow protocol | Patient/ parent health |  |  | 1 |
| Transfer of patient information | Continuity of care | Patient/ parent health |  |  | 1 |
| Transfer of patient information | Failure to follow protocol | Critical thinking |  |  | 1 |
| Transfer of patient information | Mistakes | Equipment factors |  |  | 1 |
| Transfer of patient information | Looked-after child | Continuity of care |  |  | 1 |
| Transfer of patient information | Continuity of care | Continuity of care |  |  | 1 |
| Transfer of patient information | Failure to follow protocol | Continuity of care |  |  | 1 |
| Transfer of patient information | Working conditions | Working conditions |  |  | 1 |
| Transfer of patient information | Continuity of care | Education and training |  |  | 1 |
| Transfer of patient information | Failure to follow protocol | Failure to follow protocol | Working conditions |  | 1 |
| Transport/ transfer of patients | Patient/ parent behaviour |  |  |  | 1 |
| Transport/ transfer of patients | Staff knowledge |  |  |  | 1 |
| Transport/ transfer of patients | Inadequate guidelines |  |  |  | 1 |
| Transport/ transfer of patients | Environmental |  |  |  | 1 |
| Transport/ transfer of patients | Patient health | Staff knowledge |  |  | 1 |
| Transport/ transfer of patients | Service availability | Working conditions |  |  | 1 |
| Transport/ transfer of patients | Failure to follow protocol | Education and training |  |  | 1 |
| Transport/ transfer of patients | Failure to follow protocol | Service availability | Continuity of care |  | 1 |
| Transport/ transfer of patients | Service availability | Working conditions | Working conditions |  | 1 |
| Transport/ transfer of patients | Staff knowledge | Patient age | Inadequate guidelines | Education and training | 1 |
| Treatment and procedures | Patient /parent language |  |  |  | 1 |
| Treatment and procedures | Critical thinking | Patient/ parent health |  |  | 1 |
| Treatment and procedures | Continuity of care | Patient/ parent knowledge |  |  | 1 |
| Treatment and procedures | Inadequate guidelines | Staff knowledge |  |  | 1 |
| Treatment and procedures | Environmental | Inadequate guidelines |  |  | 1 |
| Treatment and procedures | Education and training | Working conditions |  |  | 1 |
| Treatment and procedures | Patient health | Working conditions |  |  | 1 |
| Treatment and procedures | Failure to follow protocol | Working conditions |  |  | 1 |
| Treatment and procedures | Equipment factors | Working conditions |  |  | 1 |
| Treatment and procedures | Working conditions | Working conditions |  |  | 1 |
| Treatment and procedures | Mistake | Working conditions |  |  | 1 |
| Treatment and procedures | Patient age | Education and training |  |  | 1 |
| Treatment and procedures | Equipment factors | Education and training |  |  | 1 |
| Treatment and procedures | Continuity of care | Looked-after child | Patient/ parent geography |  | 1 |
| Treatment and procedures | Continuity of care | Failure to follow protocol | Patient health |  | 1 |
| Treatment and procedures | Mistake | Working conditions | Working conditions |  | 1 |
| Treatment and procedures | Failure to follow protocol | Working conditions | Education and training |  | 1 |
| Treatment and procedures | Patient age | Patient/ parent geography | Education and training | Continuity of care | 1 |
| Treatment decisions | Patient age |  |  |  | 1 |
| Treatment decisions | Staff knowledge |  |  |  | 1 |
| Treatment decisions | Mistake |  |  |  | 1 |
| Treatment decisions | Failure to follow protocol | Failure to follow protocol |  |  | 1 |
| Treatment decisions | Patient age | Inadequate guidelines |  |  | 1 |
| Treatment decisions | Patient/ parent behaviour | Inadequate guidelines |  |  | 1 |
| Treatment decisions | Staff knowledge | Inadequate guidelines |  |  | 1 |
| Treatment decisions | Patient age | Continuity of care |  |  | 1 |
| Treatment decisions | Inadequate guidelines | Continuity of care |  |  | 1 |
| Treatment decisions | Staff knowledge | Continuity of care |  |  | 1 |
| Treatment decisions | Failure to follow protocol | Staff knowledge | Patient age |  | 1 |
| Treatment decisions | Continuity of care | Inadequate guidelines | Working conditions |  | 1 |
| **Total** |  |  |  |  | **1257** |
