# Supplementary material for: Patient Safety Incidents Involving Sick Children in Primary Care in England and Wales: A Mixed Methods Analysis
Source: PLoS Med. 2017 Jan 17;14(1):e1002217. doi: 10.1371/journal.pmed.1002217 (PMC5240916; doi:10.1371/journal.pmed.1002217)
Supplement: S6 Table — (DOCX) [file pmed.1002217.s007.docx]

S6 Table: Consolidated criteria for reporting qualitative research (COREQ): a 32-item checklist

| No | Item | Guide questions/description | Response |
| --- | --- | --- | --- |
| Domain 1: Research team and reflexivity | | | |
| Personal Characteristics | | | |
| 1. | Interviewer/facilitator | Which author/s conducted the interview or focus group? | We were not involved in any data collection – no interviews or focus groups were conducted. |
| 2. | Credentials | What were the researcher's credentials? E.g. PhD, MD |  |
| 3. | Occupation | What was their occupation at the time of the study? |  |
| 4. | Gender | Was the researcher male or female? |  |
| 5. | Experience and training | What experience or training did the researcher have? |  |
| Relationship with participants | | | |
| 6. | Relationship established | Was a relationship established prior to study commencement? | We were not involved in any data collection – no interviews or focus groups were conducted. |
| 7. | Participant knowledge of the interviewer | What did the participants know about the researcher? e.g. personal goals, reasons for doing the research |  |
| 8. | Interviewer characteristics | What characteristics were reported about the interviewer/facilitator? e.g. Bias, assumptions, reasons and interests in the research topic |  |
| Domain 2: study design | | | |
| Theoretical framework | | | |
| 9. | Methodological orientation and Theory | What methodological orientation was stated to underpin the study? e.g. grounded theory, discourse analysis, ethnography, phenomenology, content analysis | We used thematic analysis. (Methods Para 8) |

| Participant selection | | | |
| --- | --- | --- | --- |
| 10. | Sampling | How were participants selected? e.g. purposive, convenience, consecutive, snowball | A purposive sample of reports was selected, (Methods Para 8) |
| 11. | Method of approach | How were participants approached? e.g. face-to-face, telephone, mail, email | We did not approach participants. |
| 12. | Sample size | How many participants were in the study? | We did not have study participants. We selected all cases fulfilling our inclusion criteria from the dataset. |
| 13. | Non-participation | How many people refused to participate or dropped out? Reasons? | We did not have study participants. Not applicable. |
| Setting | | | |
| 14. | Setting of data collection | Where was the data collected? e.g. home, clinic, workplace | Methods Para 2-3 |
| 15. | Presence of non- participants | Was anyone else present besides the participants and researchers? | N/A |
| 16. | Description of sample | What are the important characteristics of the sample? e.g. demographic data, date | We have not included these details for our qualitative sample as this was a mixed methods study and the purpose of the qualitative analyses was to supplement the quantitative findings. The characteristics of our included data however are described in the Results (Para 2). |
| Data collection | | | |
| 17. | Interview guide | Were questions, prompts, guides provided by the authors? Was it pilot tested? | We were not involved in data collection. |
| 18. | Repeat interviews | Were repeat interviews carried out? If yes, how many? |  |
| 19. | Audio/visual recording | Did the research use audio or visual recording to collect the data? |  |
| 20. | Field notes | Were field notes made during and/or after the interview or focus group? |  |
| 21. | Duration | What was the duration of the interviews or focus group? |  |
| 22. | Data saturation | Was data saturation discussed? |  |

| 23. | Transcripts returned | Were transcripts returned to participants for comment and/or correction? | We did not have study participants. |
| --- | --- | --- | --- |
| Domain 3: analysis and findings | | | |
| Data analysis | | | |
| 24. | Number of data coders | How many data coders coded the data? | 2 researchers (PR, ACS) |
| 25. | Description of the coding tree | Did authors provide a description of the coding tree? | Yes – the multi-axial framework used to classify incidents using the recursive model of incident analysis is described in detail and now included as supplemental information (S2-4 Text). We also describe how themes and sub-themes (i.e. our coding tree) were developed. |
| 26. | Derivation of themes | Were themes identified in advance or derived from the data? | They were derived from the data. *“New codes were created to capture additional semantic (descriptive and superficial) insights and latent (underlying or inferred) insights present in reports and the contexts in which incidents occurred [25, 35, 36]. These codes were grouped into themes and sub-themes (by PR and ACS) that support our understanding of the data and the underlying reasons for certain incidents [25, 35, 36].”* The final themes were agreed upon by the analysis team through consensus. |
| 27. | Software | What software, if applicable, was used to manage the data? | QSR NVivo version 9 |
| 28. | Participant checking | Did participants provide feedback on the findings? | This was not possible as all reports are submitted as anonymised reports from source organisations. |
| Reporting | | |  |
| 29. | Quotations presented | Were participant quotations presented to illustrate the themes / findings? Was each quotation identified? e.g. participant number | Examples were presented to illustrate key findings and themes – see table 4 |
| 30. | Data and findings consistent | Was there consistency between the data presented and the findings? | Yes |
| 31. | Clarity of major themes | Were major themes clearly presented in the findings? | Yes |
| 32. | Clarity of minor themes | Is there a description of diverse cases or discussion of minor themes? | Yes |
