# Supplementary material for: Patient Safety Incidents Involving Sick Children in Primary Care in England and Wales: A Mixed Methods Analysis
Source: PLoS Med. 2017 Jan 17;14(1):e1002217. doi: 10.1371/journal.pmed.1002217 (PMC5240916; doi:10.1371/journal.pmed.1002217)
Supplement: S1 Text — (DOCX) [file pmed.1002217.s008.docx]

S 1 Text: search terms used to retrieve reports involving ‘sick’ children

1. Abdo pain
2. Abdominal pain
3. Acetazolamide
4. Acute abdo
5. Anorexi
6. Antibiot
7. Apnoe
8. Appendicitis
9. Arrest
10. Arthritis
11. ASD
12. Asperg
13. Asthma
14. Ataxia
15. Atomoxetine
16. Atresia
17. Autis
18. Bacteria
19. Behavioural
20. Blanch
21. Blastoma
22. Blind
23. Blood sugar
24. Breathless
25. Bronchiolitis
26. Bronchitis
27. Burn
28. CAMHS
29. Cancer
30. Carbamazepine
31. Cardiac failure
32. Cellulitis
33. Cerebral palsy
34. Chemo
35. Citalopram
36. Clobazam
37. Clomipramine
38. Clonazepam
39. Clonidine
40. CMHT
41. Coarctation of the Aorta
42. Coelia disease
43. Coeliac disease
44. Coma
45. Congenital
46. Convulsion
47. Cough
48. Cramp
49. Crohn
50. Cyanos
51. Cystic
52. Deaf
53. Dehydrate
54. Depress
55. Deteriorate
56. Development delay
57. Diabet
58. Dialys
59. Diarrhea
60. Diarrhoea
61. Diazepam
62. Dipstick
63. Disab
64. Disorder
65. Dissociative
66. DKA
67. Down syndrome
68. Downs syndrome
69. Drowsy
70. Duloxetine
71. Dystrophy
72. E.coli
73. Ectomy
74. Eczema
75. Ehlers Danlos
76. Epilep
77. Erythema multiforme
78. [Ethosuximide](http://www.epilepsysociety.org.uk/ethosuximide)
79. Exacerbate
80. Eye syndrome
81. Foetal alcohol
82. Febrile
83. Fever
84. Floppy
85. Fluoxetine,
86. Fracture
87. Gabapentin
88. Gastritis
89. Gastritis
90. Gastroenteritis
91. GCS
92. George syndrome
93. Glucose
94. Glycosuria
95. Growth hormone
96. Haematuria
97. Haemol
98. Haemophilia
99. Haloperidol
100. Head injury
101. Headache
102. Heart
103. Hirschprung syndrome
104. HIV
105. Hydrocephalus
106. Hyper
107. Hypo
108. Imipramine
109. Immuno
110. Impetigo
111. Infect
112. Inhaler
113. Insulin
114. Intra osseous
115. Intra venous
116. Intracranial
117. Intubate
118. Intussusception
119. Irritable
120. IUGR
121. IV access
122. IV antihistamine
123. IV diazepam
124. IV fluid
125. IV sedation
126. Jaundice
127. Kawazaki disese
128. Ketoacidosis
129. Keton
130. Ketosis
131. Kidney
132. Lamotrigine
133. Learning disa
134. Leukae
135. [Levetiracetam](http://www.epilepsysociety.org.uk/levetiracetam)
136. Life saving
137. Life threatening
138. Life-saving
139. Lithium
140. Liver disease
141. Liver failure
142. Metab
143. Acido
144. Renal failure
145. Phenobarbitone
146. Sepsis
147. Lorazepam
148. lung disease
149. Lung disease
150. Lymph
151. Maln
152. Meningitis
153. Meningitus
154. Meningoco
155. Meningococcal disease
156. Methylphenidate
157. Midazolam
158. Migraine
159. Mirtazapine
160. Myopathy
161. Nebs
162. Nebu
163. Nephrotic syndrome
164. Neuroblastoma
165. [Nitrazepam](http://www.epilepsysociety.org.uk/nitrazepam)
166. Obstruct
167. Oedema
168. Olanzapine
169. Osteomyelitis
170. Otitis
171. Otitis Media
172. Overdose
173. Oxcarbazepine
174. Palsy
175. Paroxetine
176. Pendred syndrome
177. Perthes disease
178. Petech
179. [Phenobarbital](http://www.epilepsysociety.org.uk/phenobarbital)
180. Phenytoin
181. Plegia
182. Pneumococcal disease
183. Pneumonia
184. Pneumonitis
185. Polyuria
186. Promazine
187. Psychiat
188. Psychol
189. Psychosis
190. Pulse
191. Pyrexia
192. Quetiapine
193. Qunisy
194. Rash
195. Refeeding syndrome
196. Reflux
197. Renal disease
198. Renal failure
199. Renal func
200. Resp rate
201. Respiratory rate
202. Responsive
203. Retts syndrome
204. Rey’s syndrome
205. Risperidone
206. Ritalin
207. Salbutamol
208. Sats
209. Saturation
210. Scabies
211. Scalds
212. Scoliosis
213. Seizure
214. Self harm
215. Self-harm
216. Septic
217. Septic
218. Croup
219. Obstruct
220. Oedema
221. Sertraline
222. Shortness of breath
223. Sick
224. Sickle cell disease
225. Sickle disease
226. Sodium valproate
227. Spina bifida
228. Spinal muscular atrophy
229. Splenectomy
230. Squint
231. Staph
232. Steroid
233. Strattera
234. Streptococcal
235. Suicide
236. SVT
237. Swollen
238. Tachy
239. Talipes
240. Tender
241. Thalassaemia
242. Thrive
243. Thrombo
244. Thyroid
245. Tonsillitis
246. Topiramate
247. Torticollis
248. Transposition of the great arteries
249. Tuberous sclerosis
250. Tumour
251. Undescended
252. Urinary tract
253. Urticaria
254. Valproate
255. Vasculitis
256. Ventilation
257. Ventolin
258. Viral
259. Visual impairment
260. Von willebrand
261. VSD
262. Westerdrout syndrome
263. Wheeze
264. Zolpidem
