# Supplementary material for: Patient Safety Incidents Involving Sick Children in Primary Care in England and Wales: A Mixed Methods Analysis
Source: PLoS Med. 2017 Jan 17;14(1):e1002217. doi: 10.1371/journal.pmed.1002217 (PMC5240916; doi:10.1371/journal.pmed.1002217)
Supplement: S2 Text — (DOCX) [file pmed.1002217.s009.docx]

S 2 Text: incident descriptors framework

**1 ** ADMINISTRATION ****

1.1 Filing system – information filed incorrectly

1.2 Message handling – errors in the taking and distributing of messages

1.3 Appointments – errors in managing appointments for healthcare

1.3.1 Primary care appointments

1.3.2 Secondary care appointments

1.4 Payment – errors in the process of healthcare payment systems

1.5 Ability to access healthcare professional – delays or unable to see healthcare professional

1.5.1 Home visits

1.5.2 Returning phone calls

1.5.3 Out-of-hours

1.5.4 Health visiting

1.5.5 Child and Adolescent Mental Health Services

1.5.6 Occupational therapy

1.6 Transfer of patient information – incorrect or inefficient transfer of patient information across healthcare systems

1.6.1 Between care settings

1.6.1.1 From primary to secondary care

1.6.1.1.1 Lost

1.6.1.1.2 Not sent

1.6.1.1.3 Incorrect/incomplete

1.6.1.1.4 Delayed

1.6.1.1.5 Illegible

1.6.1.2 From secondary to primary care

1.6.1.2.1 Lost

1.6.1.2.2 Not sent

1.6.1.2.3 Incorrect/incomplete

1.6.1.2.4 Delayed

1.6.1.2.5 Illegible

1.6.1.3 Between primary care settings

1.6.1.3.1 Lost

1.6.1.3.2 Not sent

1.6.1.3.3 Incorrect/incomplete

1.6.1.3.4 Delayed

1.6.1.3.5 Illegible

1.6.2 New diagnoses – incorrect or inefficient transfer of patient information from secondary care regarding new diagnoses

1.6.3 Appropriate follow up – incorrect or inefficient transfer of patient regarding necessary follow-up of patient. e.g. requirements for follow up screening or regular review

1.6.4 Involving out-of-hours – incorrect or inefficient transfer of patient information between in- and out- of hours services

1.6.5 NHS direct – incorrect or inefficient transfer of patient information between NHS direct and other services

1.7 Breaches of confidentiality – patient confidentiality breached via documentation error

**2 ** DOCUMENTATION****

2.1 Medical records – errors involving patient’s personal medical records

2.1.1 Record(s) unavailable – records could not be accessed when needed

2.1.1.1 Red book

2.1.1.2 General practice records

2.1.1.3 Child health records

2.1.1.4 Lost medical records

2.1.2 Care given but not documented – records did not contain documentation of care

2.1.3 Record not up to date or complete – information missing from records

2.1.3.1 Discrepancies between vaccine records

2.1.3.1.1 Red book

2.1.3.1.2 General practice records

2.1.3.1.3 Child health records

2.1.4 Inaccurate or unclear medical records / medical record error

2.1.4.1 Red book

2.1.4.2 General practice records

2.1.4.3 Child health records

2.2 Death certificates – errors concerning patient’s death certificates

**3 ** REFERRAL ****

3.1 Human – human referral errors i.e. not system-based

3.1.1 Not performed when indicated – a person failed to refer when indicated

3.1.1.1 Delayed referral – errors in the timely referral of patients

3.1.1.1.1 Secondary care

3.1.1.1.2 Specialist care

3.1.1.1.3 Emergency care

3.1.1.1.4 Nursing

3.1.1.1.5 Social care

3.1.1.1.6 Health visitor

3.1.1.1.7 General practice

3.1.1.1.8 Child and Adolescent Mental Health Services

3.1.1.2 Referral not made when appropriate – referral decision-making error

3.1.1.2.1 Secondary care

3.1.1.2.2 Specialist Care

3.1.1.2.3 Emergency Care

3.1.1.2.4 Nursing

3.1.1.2.5 Health visitor

3.1.1.2.6 Social care

3.1.1.2.7 General practice

3.1.1.2.8 Child and Adolescent Mental Health Service

3.1.1.3 No follow up arranged – did not follow-up or were not asked to follow-up

3.1.2 Incomplete /incorrect referral – someone did not complete referral

3.1.3 Illegible referral – someone created an illegible referral letter/ document

3.1.4 Inappropriate referral to primary care – work inappropriately passed to primary care

3.1.5 Inappropriate referral – someone inappropriately referred a patient

3.1.6 Referral refused – someone refused to accept a referral of a patient

3.2 Administration

3.2.1 Not sent– letter of referral erroneously not sent by office

3.2.2 Delayed – letter of referral delayed at office level

3.2.3 Lost – letter of referral lost in the system

3.2.4 Not acted upon – referral successful but patient not seen by physic

3.2.4.1 Refused patient referral refused by receiving office

3.2.5 Inappropriate referral– referral made erroneously at office level

3.2.6 Social work referral issues –administrative errors in the social work referral process

**4 ** DIAGNOSIS AND ASSESSMENT ****

4.1 Diagnosis – errors in the process of identifying/ defining a patient’s illness

4.1.1 Missed diagnosis – failing to spot a particular illness

4.1.2 Wrong diagnosis – misidentifying the patient’s illness

4.1.3 Delayed diagnosis – not identifying an illness in a timely manner

4.1.3.1 Cancer

4.1.3.2 Emergency condition

4.1.3.3 Contagious condition

4.2 Insufficient assessment – not adequately assessing the patient clinically

4.2.1 Triage – errors in the process of assessing the severity of a patient’s condition

4.2.1.1 By healthcare professional

4.2.1.2 By non-healthcare professional

4.2.2 History – errors in the process of taking a patient’s medical history

4.2.3 Examination – errors in the process of examining patients

4.2.4 Identifying vulnerable or high–risk patient – failure to identify risky patients

4.2.5 Emergency vehicle use – inappropriate transfer vehicle used (e.g. private vehicle instead of ambulance)

4.2.6 Discharge planning – premature discharge and poor discharge planning

4.3 Delayed assessment – a delay in assessment for care or care adjunct

**5 ** TREATMENT & PROCEDURES (excludes drugs/vaccines)**

5.1 Clinical treatment decision – errors in decisions to treat or how to treat

5.1.1 No treatment given – inappropriate decision not to treat

5.1.2 Insufficient treatment given – failure to provide adequate treatment

5.1.3 Wrong treatment given – providing inappropriate treatment

5.2 Other non-medication treatment errors

5.2.1 Ordering treatments – wrong treatment ordered or treatment not ordered when appropriate

5.2.2 Implementation – error in conducting the correctly chosen process or procedure

5.2.3 Complication

5.2.3.1 Complication from execution of procedure

5.2.3.2 Adverse event suffered by patient as a result of treatment other than medication

5.2.4 Timeliness – treatment other than medication not administered in a timely fashion

5.2.5 Execution of care – error in choosing the correct process or procedure

5.2.6 Wrong anatomical side/site – administering treatment at the wrong site

5.2.7 Insufficient supply of treatment – not having adequate supplies to treat patients

**6 ** MEDICATION & VACCINES ****

6.1 Clinical treatment decision – errors in decisions to treat or how to treat with medications

6.1.1 No treatment given – inappropriate decision not to treat

6.1.2 Insufficient treatment – failure to provide adequate treatment

6.1.3 Wrong treatment given – providing inappropriate treatment

6.1.4 Treatment not ordered – failure to request an appropriate treatment

6.2 Medication prescribing – errors in the medication prescribing process

6.2.1 Wrong medication – patient was prescribed incorrect medication

6.2.2 Wrong patient – mediation was prescribed for wrong patient

6.2.3 Wrong dose – medication was prescribed at incorrect dose

6.2.4 Wrong route – medication was prescribed for incorrect route

6.2.5 Wrong time – medication was prescribed for incorrect/ inappropriate time

6.2.6 Unsafe medication – mediation prescribed was unsafe

6.2.6.1 Teratogenic

6.2.6.2 Contraindicated

6.2.6.3 Allergy

6.2.7 Wrong formulation – inappropriate formulation of medication was prescribed e.g. liquid versus tablet

6.2.8 Wrong number of doses – incorrect quantity of medication prescribed

6.2.9 Illegible/ unclear prescription – prescription document is unclear

6.2.10 Incomplete prescription e.g. brand not specified – prescription document not fully completed

6.3 Medication dispensing – errors in the medication dispensing process

6.3.1 Wrong medication – patient was dispensed incorrect medication

6.3.2 Wrong patient – medication was dispensed to incorrect patient

6.3.3 Wrong dose – medication was not dispensed at safe dose intended

6.3.4 Wrong route – incorrect dose of medication was dispensed

6.3.5 Wrong time – medication was not dispensed for appropriate time

6.3.6 Wrong formulation – medication was not dispensed as appropriate formulation e.g. liquid versus tablet

6.3.7 Not dispensed – medication was not dispensed

6.3.8 Allergy – dispensed to a patient with known allergy

6.3.9 Out of date – medication dispensed was out of date

6.3.10 Wrong label – medication was dispensed with wrong label

6.3.11 Wrong number of doses – wrong quantity of medication was dispensed

6.3.12 Inappropriate medication – medication dispensed was inappropriate e.g. for that specific patient

6.3.13 Wrong container – medication was dispensed in inappropriate container

6.4 Medication administration – errors in the medication administering process

6.4.1 Wrong medication – patient received incorrect medication

6.4.2 Wrong patient – patient received another patient’s medication

6.4.3 Wrong dose – patient received incorrect medication dose

6.4.4 Wrong route – patient received medication via the incorrect route

6.4.5 Wrong time – patient took medication at incorrect time

6.4.6 Wrong formulation – patient took inappropriate medication formulation

6.4.7 Out of date – patient took out of date medication

6.4.8 Allergy – patient received medication they had a known allergy to

6.4.9 Medication not administered – patient did not receive medication

6.4.10 Reconstitution error – patient received inappropriately reconstituted medication

6.5 Monitoring medication – error in the process of monitoring dose-dependent medications, or those with side effects

6.5.1 Lack of monitoring – failure to appropriately monitor

6.5.2 Medication dose not appropriately adjusted – failed to appropriately act on monitoring

6.6 Adverse event – patient suffered a complication as a result of medication

6.6.1 Allergy – unknown that patient had any allergies

6.7 Drug omission – medication was erroneously not given to or not taken by patient

6.8 Patient overdose – patient self-administered overdose

6.9 Incorrect storage of medication – medication was not stored appropriately

6.10 Medication timeliness – medication was not commenced in a timely fashion

6.11 Vaccines

6.11.1 Vaccine prescribing – errors in the vaccine prescribing process

6.11.1.1 Wrong vaccine – patient was not prescribed appropriate vaccine

6.11.1.2 Wrong patient – vaccine was prescribe for wrong patient

6.11.1.3 Wrong dose – vaccine was not prescribed at appropriate dose

6.11.1.4 Wrong route – vaccine was not prescribed for appropriate route

6.11.1.5 Wrong time – vaccine was not prescribed for appropriate time

6.11.1.6 Contraindicated – vaccine prescribed was contraindicated

6.11.1.7 Wrong formulation – vaccine prescribed was of wrong formulation

6.11.1.8 Wrong number of doses – incorrect quantity of vaccines prescribed

6.11.2 Vaccine dispensing – errors in the vaccine dispensing process

6.11.2.1 Wrong vaccine – patient was not dispensed appropriate vaccine

6.11.2.2 Wrong patient – vaccine was dispensed for wrong patient

6.11.2.3 Wrong dose – vaccine was dispensed at incorrect dose

6.11.2.4 Wrong route – vaccine was dispensed for incorrect route

6.11.2.5 Wrong time – vaccine was not dispensed for inappropriate time

6.11.2.6 Wrong number of doses – incorrect quantity of vaccines were dispensed

6.11.2.7 Stored incorrectly – vaccines were not stored correctly

6.11.2.8 Out of date – expired vaccines were in storage

6.11.2.9 Not dispensed – vaccines were unavailable/ not dispensed

6.11.2.10 Wrong formulation – incorrect vaccine formulations were stored

6.11.2.11 Wrong label – vaccines were dispensed with incorrect labels

6.11.2.12 Contraindicated – contraindicated vaccine was dispensed

6.11.3 Vaccine administration – errors in the vaccine administering process

6.11.3.1 Wrong vaccine – patient received incorrect vaccine

6.11.3.2 Wrong patient – patient received another patient’s vaccine

6.11.3.3 Wrong dose – patient received the incorrect vaccine dose

6.11.3.4 Wrong route – patient vaccinated via incorrect route

6.11.3.5 Wrong time – patient vaccinated at incorrect time

6.11.3.6 Wrong amount – patient vaccinated with wrong number of doses

6.11.3.7 Stored incorrectly – patient vaccinated with inappropriately stored vaccine

6.11.3.8 Out of date – patient vaccinated with expired vaccine

6.11.3.9 Contraindicated vaccine – patient vaccinated with contraindicated vaccine

6.11.3.10 Not administered – patient not vaccinated

6.11.3.11 Used/dirty needle – patient vaccinated using non-sterile needle

6.11.3.12 Wrong site – patient vaccinated at wrong anatomical location

6.11.3.13 Reconstitution error – patient vaccinated with inappropriately reconstituted vaccine

6.11.4 Adverse event – patient suffered a complication as a result of medication

6.11.5 Batch recall – a batch of vaccines recalled after use

6.12 Vaccine unavailable – unable to source appropriate vaccines

**7 ** INVESTIGATIONS ****

7.1 Laboratory – errors in the process of laboratory investigations

7.1.1 Ordering – wrong test ordered or test not ordered when appropriate

7.1.2 Implementing – errors in the process of obtaining or processing a laboratory specimen

7.1.2.1 Mislabeled sample

7.1.3 Reporting – error in the process of physician receiving accurate test results including errors of delay

7.1.4 Responding to results – inappropriate response to a laboratory result

7.2 Diagnostic imaging – errors in the process of diagnostic imaging investigations

7.2.1 Ordering – wrong test ordered or test not ordered when appropriate

7.2.2 Implementing – errors in the process of obtaining or processing of a diagnostic image

7.2.2.1 Mislabeled request form

7.2.3 Reporting – error in the process of physician receiving accurate test results including errors of delay

7.2.4 Responding to results – inappropriate response to a laboratory result

7.3 Other investigations – errors in the process of other investigations

7.3.1 Ordering – wrong test ordered or test not ordered when appropriate

7.3.2 Implementing – errors in the process of obtaining or processing of other diagnostic investigation

7.3.3 Reporting – error in the process of physician receiving accurate test results including errors of delay

7.3.4 Responding to results – inappropriate response to a result of other investigations

**8 ** COMMUNICATION ****

These are human failures, and do not include breakdowns in the systems that are used to communicate information.

8.1 With patients or caregivers – errors in communication between physicians or healthcare professionals and patients or caregivers

8.1.1 Wrong advice given to patient or caregiver – includes information about accessing emergency services, self–management or safety netting

8.1.1.1 By healthcare professional

8.1.1.2 By non–healthcare professional

8.1.2 Failure to convey seriousness/urgency of patient condition

8.1.3 Consent errors – errors in the process of obtaining informed consent

8.2 Between healthcare professionals – errors in communication between healthcare professionals

8.2.1 Failure to convey seriousness/urgency of patient condition

8.2.2 Handover–related inadequacies

8.3 Between healthcare and non–healthcare professionals

**9 ** EQUIPMENT ****

9.1 Therapeutic adjunct provision – failures in the process of therapeutic adjunct provision

9.2 Insufficient supply – failure to adequately supply equipment or a lack of equipment

9.2.1 Stolen equipment

9.3 Failure of equipment – equipment failing to fulfill its purpose

9.3.1 Damaged

9.3.2 Faulty

9.3.3 Misused

9.3.4 Computerized Physician Order Entry

**10 ** OTHER ****

10.1 Professionalism

10.2 Environmental hazard

10.3 Transport issues

10.4 Failure to prevent fall/injury

10.5 Failure to follow up ‘unwell’ or vulnerable child

10.6 Failure to prevent pressure ulcer
