# Supplementary material for: Patient Safety Incidents Involving Sick Children in Primary Care in England and Wales: A Mixed Methods Analysis
Source: PLoS Med. 2017 Jan 17;14(1):e1002217. doi: 10.1371/journal.pmed.1002217 (PMC5240916; doi:10.1371/journal.pmed.1002217)
Supplement: S3 Text — (DOCX) [file pmed.1002217.s010.docx]

S3 Text: contributory factors framework

**1 ** PATIENT OR CAREGIVER FACTORS ****

1.1 Geography - the area where patients live including its characteristics

1.1.1 Out of area - patient new to area

1.1.2 Access difficulties because of geography

1.2 Language - patient or caregiver unable to communicate in English

1.3 Behavior – the way in which patients or caregivers act of conduct themselves

1.3.1 Non-compliance - patient does not follow advice or instructions

1.3.1.1 Takes own discharge

1.3.1.2 Patient does not take medication as instructed or advised

1.3.1.3 Non-disclosure

1.3.1.4 Violent

1.4 Health - factors related to the patient's physical and mental health

1.4.1. Frailty - reduced physiological reserve, fragile

1.4.2. Disability

1.4.3. Allergy

1.4.4 Immunocompromised

1.4.5 Coagulation problems

1.4.6 Pregnancy

1.4.7 Epilepsy

1.4.8 Poor renal function

1.5. Knowledge – insufficient knowledge of inadequate application of knowledge

1.6. Looked-after child – child not in the care of their parents e.g. foster care

1.7 Age – child-specific factors

1.7.1 Weight-based dosing

1.8 Ethnicity – the child belongs to a certain social group

**2 ** STAFF FACTORS ****

2.1 Health - physical and mental wellbeing

2.1.1 Fatigue

2.2 Task - a piece of work to be done or undertaken.

2.2.1 Failure to follow protocol – not adhering to organizational guidelines

2.2.1.1 New protocol

2.2.2 Inadequate skill set/knowledge – insufficient knowledge of inadequate application of knowledge

2.3 Cognitive - includes abilities such as perception, learning, memory, language, concept formation, problem solving, and thinking.

2.3.1 Mistake – unintentional cognitive lapses

2.3.1.1 Distraction/ inattention/ oversight/forgot

2.3.1.2 Similar medication names / appearances confused

2.3.1.3 Similar patient names

2.3.1.4 Haste/ poor time management

2.3.1.5 Misread/ did not read

2.3.1.6 Patient ID label

2.3.2 Violation - deliberate breaking of a rule

2.3.3 Stress - mental or emotional strain

2.3.4 No or poor supervision or assistance of staff

2.3.5 Critical thinking – problem solving

**3 ** EQUIPMENT / MEDICATION/ VACCINE FACTORS ****

3.1 Poor design - impractical or in some way inadequate

3.2 Poor storage – impractical or inadequate storage

3.3 Poor packaging – impractical or inadequate storage

3.4 Failure of equipment/ medication/ vaccine – unable to fulfill its purpose

**4 ** ORGANIZATION FACTORS ****

4.1 Protocols or guidelines – existing guidelines not fit for purpose

4.1.1 Mental health

4.1.2 Vulnerable patients

4.1.3 Investigations

4.1.4 Referrals

4.1.5 Epilepsy management plan

4.1.6 Asthma management plan

4.1.7 School care plan

4.1.8 Diabetic management plan

4.1.9 Palliative care plan

4.2 Interpreter services - communication aids to reduce language barriers

4.3 Continuity of care – issues with the co-ordination of services

4.3.1 Patient unknown to staff

4.3.2 Within primary care

4.3.2.1 Out-of-hours service

4.3.2.2 Registering with a general practice

4.3.3 Between secondary and primary care

4.3.4 Access block - cannot move a patient because there is no space

4.3.5 Locum/ agency staff

4.4 Working conditions – factors relating to the work environment

4.4.1 Staffing levels

4.4.1.1 Shift pattern

4.4.1.2 Insufficient numbers of staff

4.4.1.2.1 Doctors

4.4.1.2.2 Nurses

4.4.1.2.3 Allied health professionals

4.4.1.3. Sickness

4.4.2 Team factors

4.4.2.1 Culture

4.4.2.2 Inadequate leadership

4.4.2.3 Disagreement amongst teams

4.4.3 Busy/overloaded by work

4.4.4 interruptions

4.5. Education and training – insufficient education and training of staff

4.5.1 Supervision

4.5.2 Knowledge of others roles

4.5.3 Caregiver training

4.6 Service availability – a required service is unavailable

4.7 Long wait for service – unacceptable delays in service access

**5 ** ENVIRONMENTAL FACTORS ****

5.1 Care facility has poor access for emergency vehicles
