# Supplementary material for: Patient Safety Incidents Involving Sick Children in Primary Care in England and Wales: A Mixed Methods Analysis
Source: PLoS Med. 2017 Jan 17;14(1):e1002217. doi: 10.1371/journal.pmed.1002217 (PMC5240916; doi:10.1371/journal.pmed.1002217)
Supplement: S4 Text — (DOCX) [file pmed.1002217.s011.docx]

S4 Text: incident outcomes framework

1 ** PATIENT HARM ** - direct harm to the patient physically or mentally

**1.1 Clinical harm – impaired bodily function**

1.1.1 Pain / discomfort

1.1.2 Swelling

1.1.3 Rash

1.1.4 Nausea

1.1.5 Redness

1.1.6 Bruising

1.1.7 Dizziness/ faint/ loss or altered consciousness

1.1.8 Bleeding

1.1.9 Changes in physiological parameters

1.1.9.1 Fever

1.1.9.2 Breathless

1.1.10 General deterioration/progression of condition

1.1.11 Pressure ulcer

1.1.11.1 Pressure ulcer developed

1.1.11.2 Pressure ulcer deteriorated

1.1.12 Other wound/ulcer

1.1.13 Admitted to the high dependency or intensive care unit

1.1.14 Seizures

1.1.15 Admitted to hospital/ visited emergency department

1.1.16 Infection

1.1.17 Migraine

1.1.18 Poor diabetic control

1.1.18.1 Diabetic ketosis/ ketoacidosis

1.1.19 Developmental delay

1.1.20 Diarrhea

1.1.21 Emergency surgery

1.1.22 Liver failure

1.1.23 Constipation

**1.2 Injury** - tissue damage

1.2.1 Laceration

1.2.2 Perforation

1.2.3 Fracture

1.2.4 Skin tear

1.2.5 Pain / discomfort

1.2.6 Swelling

1.2.7 Redness

1.2.8 Bruising

1.2.9 Bleeding

1.2.10 Needle stick

1.2.11 Burn

1.2.12 Fall

**1.3 Psychological / emotional distress – patient suffering**

**1.4 Death – the end of life**

**1.5 Cardio-respiratory arrest – inadequate circulation due to sudden cardiac failure and abnormal or absent breathing**

**2 ** PATIENT INCONVENIENCE ** - increased patient burden**

2.1 Repeated tests / procedure / additional treatment

2.2 Delays in management (assessment or treatment)

2.3 Increased documentation

2.4 Financial implication

2.5 Repeated visits to/from health care providers

2.6 Unnecessary treatment

2.7 Extended hospital stay

2.8 Hospital admission

**3 ** ORGANIZATIONAL INCONVENIENCE ** - increased organizational burden**

3.1 Increased documentation

3.2 Phone calls/follow-up

3.3 More equipment / supplies used

3.4 Delays in using facilities

3.5 Legal implication

3.6 Complaint made

3.7 Financial implication
