# Supplementary material for: Patient Safety Incidents Involving Sick Children in Primary Care in England and Wales: A Mixed Methods Analysis
Source: PLoS Med. 2017 Jan 17;14(1):e1002217. doi: 10.1371/journal.pmed.1002217 (PMC5240916; doi:10.1371/journal.pmed.1002217)
Supplement: S5 Text — (DOCX) [file pmed.1002217.s012.docx]

1. **Overview of Master of Philosophy (MPhil) thesis**

The MPhil thesis will consist of two phases of work:

- A literature review of care quality issues facing children in primary care.
- A secondary analysis of National Reporting and Learning system data in order to characterise:
  - vaccine-related safety incidents; and
  - patient safety incidents involving unwell children.

1. **Overview of literature review**
   1. Review questions

A literature review will be undertaken to answer the following questions:

- What patient safety and care quality problems are described in the published and unpublished literature for children in primary care in the UK?
- What solutions and quality improvement interventions have been proposed to improve the safety of primary care for children?
  1. Method

Searches of Ovid MEDLINE(R), Ovid EMBASE, EBSCO CINAHL, and Web of Knowledge will be conducted. Reference lists from included articles will be screened for articles that may have been missed by the electronic search. Articles of all evidence types: Randomised-controlled trials, cohort studies, cross-sectional studies, case controlled studies and case reports. The review will focus on studies aiming to identify patient safety and care quality issues in children (aged under 18) in UK primary care and potential interventions to improve them.

Results from the searches will be imported into Endnote (X7) and duplicates removed. The titles and abstracts of publications obtained by the search will be scanned and irrelevant studies removed. The full text of remaining articles will be retrieved reviewed for inclusion. Data will be extracted relating to: study design, patient characteristics and numbers, methods used, incident type, incident outcome, intervention, effect of intervention, and strengths and limitations of the article. Extracted data will undergo a thematic analysis to summarise the types of care quality issues and patient safety incidents occurring in children in UK primary care. The types and effect of improvement interventions to address these issues will be tabulated to inform recommendations to improve problem areas identified in subsequent NRLS data analysis.

1. **Overview of secondary analysis of NRLS data**
   1. Vaccine-related safety incidents

Around 14 million vaccination error opportunities exist for children receiving routine vaccinations in England and Wales each year. The Confidential Enquiry into Maternal and Child Health in primary care highlight failure to vaccinate as a significant source of death in children in 2006.

Aim: To characterise patient safety incidents, involving childhood vaccination, from primary care.

Objectives:

1. Describe the frequency of different types of reported patient safety incidents
2. Describe incident and patient characteristics such as patient age, vaccine name, potential contributory factors, incident outcomes and the severity of harm
3. Undertake a thematic analysis of a purposive sample of reports
4. Combine insights from both the quantitative and qualitative findings
   1. Patient safety incidents involving sick children

Failure to recognise acutely sick children and poor management of long-term conditions has been highlighted as a priority area for improvement by the Royal College of Paediatrics and Child Health. Primary care (first access services) has a significant role to play in these areas of concern, but little is known about the specific sources of harm to children in primary care or how these can be addressed.

Aim*:* To characterise patient safety incidents involving sick children in primary care.

Objectives:

1. Describe the frequency of different types of reported patient safety incidents
2. Describe incident and patient characteristics such as patient age; medications involved in incidents; patient diagnoses, signs or symptoms; potential contributory factors; incident outcomes; and severity of resultant harm
3. Undertake a thematic analysis of a purposive sample of reports
4. Combine insights from both the quantitative and qualitative findings
   1. Methods for secondary analysis
      1. Existing study protocol

The coding and analytical plan will adhere to the approved protocol for the NIHR HS&DR (12/64/118) funded Primary Care Patient Safety (PISA) study to characterise patient safety incident reports from general practice.^1^ The protocol has been extensively reviewed by international methodologists with quantitative, qualitative and mixed methods expertise, and two rounds of review by a NIHR funding committee prior to approval of funding.

Given the focus of this MPhil thesis, I will need to undertake additional processes to identify the relevant study population. I will detail in the subsequent sections the intended approach for data mining (3.3.2.), highlight which additional data variables will be needed for this study (3.3.3.), and intended process for thematic analysis (3.3.4.).

- - 1. Sample selection

Incidents reported to the NRLS between 2003-2013 will be identified. All reports specified as involving children will be identified through application of a manual age filter. Given the focus of the study – sick children – free-text searches will be run to identify reports of incidents involving sick children. A list of key terms and permutations pertinent to sick children will be developed, corroborated with clinicians, piloted, and iterated for maximum recall of reports involving sick children. The list of key terms will be informed by a previous analysis and the ICD-10 classification.

- - 1. Data processing (with framework for quantitative analysis)

The free-text components of identified reports will be analysed for incident type, contributor factors, severity of harm and incident outcomes as as per the PISA study. Additional data needed for this study will be:

- Vaccines or medications involved in incidents (Children’s BNF)
- Diagnoses, signs or symptoms (ICD- 10)

In addition, reports that provide new insights, or support or contradict theories emerging during classification will be noted.

Incidents, contributory factors and outcomes will be coded using the Australian Patient Safety Foundation Recursive Model for Incident Analysis.

The processed data will undergo descriptive analysis to illustrate the frequency distribution of variables including: age, time, incident types, contributory factors, severity of harms, outcomes, types of conditions and medications or vaccines implicated in incidents. Relationships between these variables will explored using cross-tabulations for example: incident type by age, incident type by contributory factors, primary incident type by contributory incidents, contributory factor one by contributory factor two, incident type by diagnosis, incident type by harm, incident type by outcome *etc*.

The relationship between the primary incident types and contributory incidents, primary incident types and contributory factors, and contributory factors with each other will be examined to identify frequently occurring clusters of codes.

- - 1. Thematic analysis

The purposive sample of reports will be imported into NVIVO 9 (QSR International). Reports will be read for familiarisation and broad brush coding will be used for orientation to the data. Reports will be coded inductively in an inclusive and descriptive manner to preserve the reports’ meaning. These codes will scrutinised and reports will be re-coded to capture both descriptive and latent insights. The codes will analysed and grouped into themes and sub-themes.

3.3.5 Mixed methods synthesis of results

Data classification will guide qualitative sampling and subsequent analysis. Quantitative and qualitative analyses will therefore be done sequentially. Priority (weighting) will be given to the quantitative data to provide insight into the commonly reported issues, and the purpose of the qualitative insights will be to supplement such insights and provide additional perspectives on the data.

The qualitative findings will be compared to and linked with the quantitative findings; this mixed methods approach should provide additional nuanced insights that would otherwise be missed.

There are no ‘a priori’ theories informing the design of this research, it will be largely inductive.

- 1. **Ethical considerations**

Given the anonymized nature of the data, Aneurin Bevan University Health Board research risk review committee waived the need for ethics review (ABHB R and D reference number SA/410/13)

- 1. **Timeline**

**Completed to Date**

- All vaccine incidents classified
- Familiarised with NVIVO
- Methods chapter drafted

**October**

- Write introduction chapter
- Thematic analysis of purposive sample of vaccine incidents
- Mixed methods analysis of vaccine incidents

**November**

- Write vaccine result chapter
- Classify incidents involving sick children

**December**

- Finish classifying incidents involving sick children
- Finish writing vaccine results chapter
- Begin literature review

**January**

- Write discussion for vaccine chapter
- Thematic analysis of purposive sample of incidents involving sick children

**Feb**

- Mixed methods analysis of incidents involving sick children
- Write results chapter for safety incidents involving sick children

**March**

- Sick children discussion chapter
- Synthesise literature review findings
- Write literature review chapter

**April**

- Prepare appendices
- Check references
- Check formatting

**Reference**

1. NIHR. HS&DR 12/64/118: Characterising the nature of primary care patient safety incident reports in England and Wales: mixed methods study 2013 [cited 2014 1st Sep]. Available from: http://www.nets.nihr.ac.uk/projects/hsdr/1264118.
